# ﻿What is underneath? – UV analysis of wing undersides reveals high intraspecific variability in cryptic *Agrodiaetus* species (Lepidoptera, Lycaenidae, *Polyommatus*)

**DOI:** 10.3897/zookeys.1256.165602

**Published:** 2025-10-20

**Authors:** Maria S. Vishnevskaya, Vladimir A. Lukhtanov, Alexander V. Dantchenko, Elizaveta Yu. Gorodilova

**Affiliations:** 1 Department of Entomology, Saint Petersburg State University, Universitetskaya emb. 7-9-11, Saint Petersburg 199034, Russia Saint Petersburg State University Saint Petersburg Russia; 2 Zoological Institute, Russian Academy of Sciences, Universitetskaya emb. 1, Saint Petersburg 199034, Russia Zoological Institute, Russian Academy of Sciences Saint Petersburg Russia; 3 Centre for Molecular and Cell Technologies, Research Park, Saint Petersburg State University, Botanicheskaya str. 17, Peterhof 198504, Russia Saint Petersburg State University Peterhof Russia

**Keywords:** Cryptic species, Lepidoptera, monomorphic complex, subgenus *Agrodiaetus*, ultraviolet spectrum, UV analysis, UV pattern

## Abstract

While most butterfly species in the genus *Polyommatus* Latreille, 1804 (Lepidoptera, Lycaenidae) exhibit strong sexual dimorphism, several species in the subgenus Agrodiaetus Hübner, 1822 are monomorphic, with males and females having a similar brown coloration. It is also known that this monomorphic complex of *Agrodiaetus* butterflies consists of morphologically very similar cryptic species that are almost identical in coloration and wing pattern, at least to the human eye, which, unlike that of butterflies, does not perceive ultraviolet (UV) light. However, these species can be reliably distinguished by karyotype and molecular markers. In this article it is demonstrated that cryptic species of the monomorphic complex are quite variable in wing pattern coloration in the UV spectrum. Unexpectedly, a very high level of intraspecific morphological variability was found, which concerns the degree of UV reflection and absorption by the wings or their parts, as well as the presence or absence of various elements of the wing pattern. This variability far exceeds those few variations of a single plan that is seen in the visible spectrum. At the same time, it is important to note that all these differences are intraspecific rather than interspecific; that is, the butterflies are morphologically polymorphic, but the species themselves do not differ. In this work 13 types of UV pattern are described, their distributions analysed across species and populations as well as differences in UV pattern between allopatric and sympatric populations. Variability in UV coloration in butterflies within the *Agrodiaetus* monomorphic complex and its probable relation to caterpillar food preferences are discussed.

## ﻿Introduction

Significance of wing coloration and pattern in Lepidoptera is beyond doubt. It is known to be important in mimicry, aposematism, mate recognition, and intra- and interspecific mate discrimination (e.g. Obara 1970; Benson 1972; Meyer-Rochow and Eguchi 1983; Vane-Wright and Ackery 1984; Matsuno 1988; Bernard and Remington 1991; Vane-Wright and Boppre 1993; Uésugi 1996; Jiggins et al. 2001; Fordyce et al. 2002; Nosil et al. 2003; Kemp 2007; Imafuku et al. 2007; Imafuku 2008; Kemp and Rutowski 2011; Bálint et al. 2012). Conspecific recognition and the phenomenon of interspecific discrimination usually result in noticeable morphological differences between species. However, in some cases, closely related species of Lepidoptera are cryptic, that is, morphologically indistinguishable or almost indistinguishable. The existence of cryptic species raises the questions: (1) how do morphologically identical species of butterflies choose conspecific partner during mating or (2) do cryptic species have cryptic morphological traits which are invisible to a human eye? Here we try to answer one of these questions on the example of butterflies from the monomorphic complex of the subgenus Agrodiaetus Hübner, 1822.

The subgenus Agrodiaetus includes numerous cryptic species, which differ in cytogenetic and molecular traits being morphologically similar (e.g. de Lesse 1960a, 1960b, 1961, 1962; Wiemers 2003; Kandul et al. 2004, 2007; Lukhtanov et al. 2005, 2015; Vila et al. 2010; Stradomsky and Fomina 2013; Lukhtanov and Tikhonov 2015; Shapoval and Lukhtanov 2015a, 2015b). The vast majority of cryptic species is concentrated in the so-called monomorphic species complex. This complex includes butterflies from two sister clades, *P. dolus* (Hübner, 1823) and *P. admetus* (Esper, 1783), which were revealed by the analysis of two mitochondrial markers, COI and COII (see Kandul et al. 2004). Unlike the other representatives of the subgenus, in this complex both males and females have the same brown coloration of the upperside of the wings except for *P. dolus*, *P. menalcas* (Freyer, 1837) and *P. fulgens* (de Sagarra,1925).

At present, the studies of monomorphic species complex of the subgenus Agrodiaetus are aimed at using molecular markers and karyotypes to delimit species and reveal hidden species diversity within the group (e.g. Lukhtanov and Dantchenko 2002a, 2024; Wiemers 2003; Kandul et al. 2004, 2007; Lukhtanov et al. 2005, 2006; Kolev 2005; Dincă et al. 2013, 2017; Vishnevskaya et al. 2016, 2018). These genetically unlinked markers serve as reliable traits for making taxonomic hypotheses and provide indirect evidence for the absence or presence of interspecific hybridization in this group (Lukhtanov 1989; Kandul and Lukhtanov 1997; Lukhtanov et al. 1998, 2006, 2008, 2015; Lukhtanov and Dantchenko 2002a, 2002b, 2003; Lukhtanov and Budashkin 2007; Vila et al. 2010; Vishnevskaya et al. 2016, 2018).

In our previous studies, we tried to find tiny differences in wing coloration of the monomorphic butterflies, which resulted in description of different types of wing pattern. However, these types of wing pattern were not correlated with the molecular and chromosomal data and were randomly distributed across the molecular phylogeny (Vishnevskaya et al. 2016). In 2022 an article on *P. aroaniensis* Brown, 1976 species complex appeared, where the authors, using an integrative approach, described a new species *P. lurae* Parmentier, Vila & Lukhtanov, 2022 (Parmentier et al. 2022). In addition to standard molecular and cytogenetic methods, the authors used morphometry to distinguish between species. The authors argued that the new taxon is ecologically specialized, inhabiting dark-colored soils, which may explain its different coloration compared to the other taxa of *P. aroaniensis* species group (Parmentier et al. 2022). Their study was conducted in the visible spectrum. Within the present article we extended this analytical framework by analyzing wing patterns in the ultraviolet spectrum.

Some authors point the presence of certain patterns of wing coloration in the invisible ultraviolet spectrum, interpreting this as a signal that promotes intra- and interspecific communication (e.g. Mazokhin-Porshniakov 1957; Nekrutenko 1964; Silberglied and Taylor 1973; Silberglied 1979; Meyer-Rochow 1991; Vane-Wright and Boppre 1993; Brunton and Majerus 1995; Kemp 2008; Imafuku 2008; Obara et al. 2008, 2010).

Previous research (Lukhtanov et al. 2005) did not find any differences in the UV spectrum among species of the monomorphic complex, as the upperside of the wings almost completely absorbs UV light). In this study, we discovered that the underside of the butterflies’ wings can reflect UV light and demonstrates high variability in UV pattern. For the brown *Agrodiaetus* butterflies this was an unexpected finding, since the butterflies in the monomorphic complex are nearly identical in visible light.

Within this study, our objective was to analyze hidden patterns on the underside of the wings using UV imaging. First, we tried to find fixed species-specific characters. Second, we examined correlation of the patterns with the taxonomy and the level of divergence. Third, we analyzed correlation of the pattern with geography, i.e. interspecific and intraspecific variability in different parts of the area (for which we have separately analyzed butterflies from Azerbaijan, Armenia, Turkey, Iran, Russia, Balkan Peninsula). Finally, we analyzed differences in the wing pattern between allopatric and sympatric populations (in order to understand whether hidden pattern could be used as a signal for recognition of a conspecific partner within species with overlapping areas).

## ﻿Materials and methods

For our study we used *Agrodiaetus* species from the monomorphic complex collected in different locations during the time period of 2001–2024 (see Suppl. material 1). Altogether, 294 butterfly specimens were studied (247 males and 47 females), and the UV images were obtained for these samples. Most of them (238 specimens) were identified based on molecular and/or chromosomal analysis. Molecular and cytogenetic data on most of the material has already been described in previous research (Lukhtanov et al. 2003, 2005, 2015; Vershinina and Lukhtanov 2010; Vishnevskaya et al. 2016; Lukhtanov and Dantchenko 2024). For some of the specimens molecular and chromosome data were obtained within this study and are presented for the first time, including the data on the butterflies collected in Azerbaijan during the expedition in 2024. In some cases where there was no doubt, the specimens were identified based on morphology. All this information is noted in Suppl. material 1.

### ﻿Butterfly preparation and molecular analysis

Before processing butterflies were put in glassine envelopes and kept alive for less than one hour. The abdomen was removed and fixed in a fresh fixative (3:1, 96% ethanol: glacial acetic acid) for further chromosomal analysis. The butterflies were kept dry before DNA-extraction processing.

To distinguish between species in new material we used standard mitochondrial DNA barcode (657 bp length) which was obtained for 85 samples. The DNA was extracted from new butterflies as described in Vishnevskaya et al. (2016). Chromosome preparation techniques used for the new material are also described in Vishnevskaya et al. (2016). All the preparations were held in “Chromas” Core Facility, Research Park, Saint Petersburg University. Sequencing was held in Research Resource Centre for Molecular and Cell Technologies, Research Park, Saint Petersburg University. The obtained sequences were aligned and compared using BIOEDIT v. 7.2.5. Information on sequences (GenBank ID) is present in Suppl. material 1.

### ﻿Ultraviolet photography

In our research we paid special attention to studying the wing coloration in the UV spectrum invisible to human eye. For the analysis we used wings separated from the body as well as several pinned butterfly specimens.

To analyze the wing coloration in the UV spectrum, we used methods based on the ones described in the articles by Meyer-Rochow (1991) and Pecháček et al. (2019), with modifications for specific objects and tasks of the current study: the diaphragm closed; the exposition of 15 seconds; the ISO set at 400. In order to detect UV patterns on the wings of butterflies (male and female), photographs of the wings were taken from the upperside and from the underside. As a source of ultraviolet we used UV lamps, emitting light at a wavelength of 320–380 nm (which matches with the maximum sensitivity of photoreceptors sensitive to ultraviolet radiation in the eyes of insects; Meyer-Rochow 1991). Photographs were taken with a Canon camera (EOS 1100 D) with a modified sensor. The camera was supplied with a lens that transmits UV light (Kyoei Kuribayashi, 35mm F/ 3.5), supplemented with a filter that cuts off visible light from 380 nm for shooting in UV (Baader U-Filter, 320–380 nm, Baader Planetarium, Germany). Before the analysis images were processed in PHOTOSHOP v. 23.4.1. to make the color balance equal in all images. Then the images were converted to grayscale.

### ﻿Images of the wings in visible spectrum

Images of upper and underside of the wings in the visible spectrum were obtained using the scanner Epson Perfection 4870 Photo. The images of several pinned butterflies were taken with the Canon EOS 5D Mark IV camera, a Laowa 100mm f/2.8 2X Ultra Macro APO lens and a Canon Macro Twin Lite MT-26EX-RT flash.

## ﻿Results

### ﻿UV pattern types

We analyzed 294 specimens (Table 1) from 16 species of the monomorphic complex of the subgenus Agrodiaetus (Suppl. material 1). The taxa representing *admetus* clade included 232 samples, while of the *dolus* clade included 62 samples. All the photographs of wing coloration of the species in visible and UV light are present in Suppl. materials 2–28.

**Table 1.** Total number of butterfly specimens in the research.

Clade	Species name	Number of specimens in each species sample	Number of females in each species sample
* admetus *	*P. admetus*	43	11
	*P. demavendi*	56	0
	*P. emmeli*	10	0
	*P. keleybaricus*	5	0
	*P. khorasanensis*	9	0
	*P. nephohiptamenos*	7	0
	*P. pseudorjabovi*	71	25
	*P. ripartii*	31	1
* dolus *	*P. alcestis*	2	0
	*P. aroaniensis*	1	0
	*P. dantchenkoi*	5	0
	*P. eriwanensis*	4	0
	*P. orphicus*	22	8
	*P. rjabovianus*	16	2
	*P. timfristos*	6	0
	*P. valiabadi*	6	0

Samples of monomorphic species complex showed no difference in the upperside of the wings in the UV light. They did not reflect the UV light resulting in black color in the photographs. Therefore, in the analysis we used only photographs of UV patterns of the underside of the wings.

**Figure 1.**
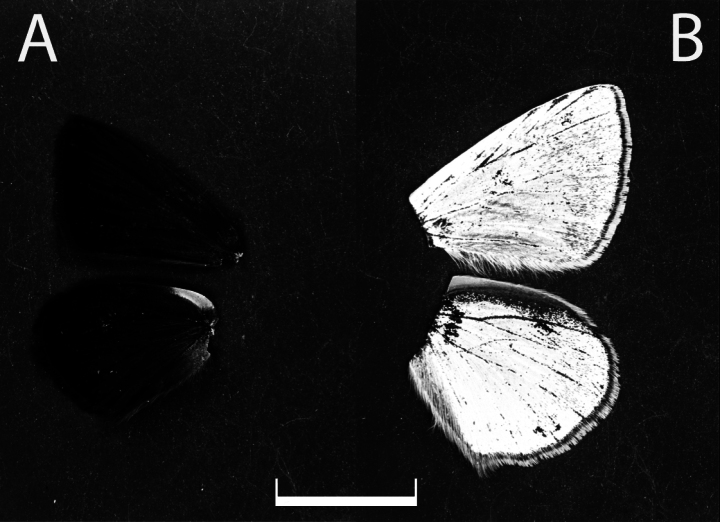
Upperside of Polyommatus (Agrodiaetus) wings in UV light. A. *P. orphicus orphicus* (PE016, female) (brown in visible color); B. *P. hamadanensis* (N500, male) (violet-blue in visible light). Scale bar: 1 cm.

The underside of the *Agrodiaetus* butterfly wing can reflect and partially or absolutely absorb the UV light with different degree. To make it more clear what an absolute reflectance and absolute absorption is, we provide a photograph of the blue butterfly male with the blue upperside color (*Polyommatus hamadanensis* de Lesse, 1959) and the butterfly from the brown-species complex (*P. orphicus orphicus* Kolev, 2005) (Fig. 1).

The underside of the wings varied greatly between species and within species showing different variations of the pattern of UV light reflectance and absorption. We use the word “pattern” since within a single wing one can see reflecting and absorbing areas.

**Figure 2.**
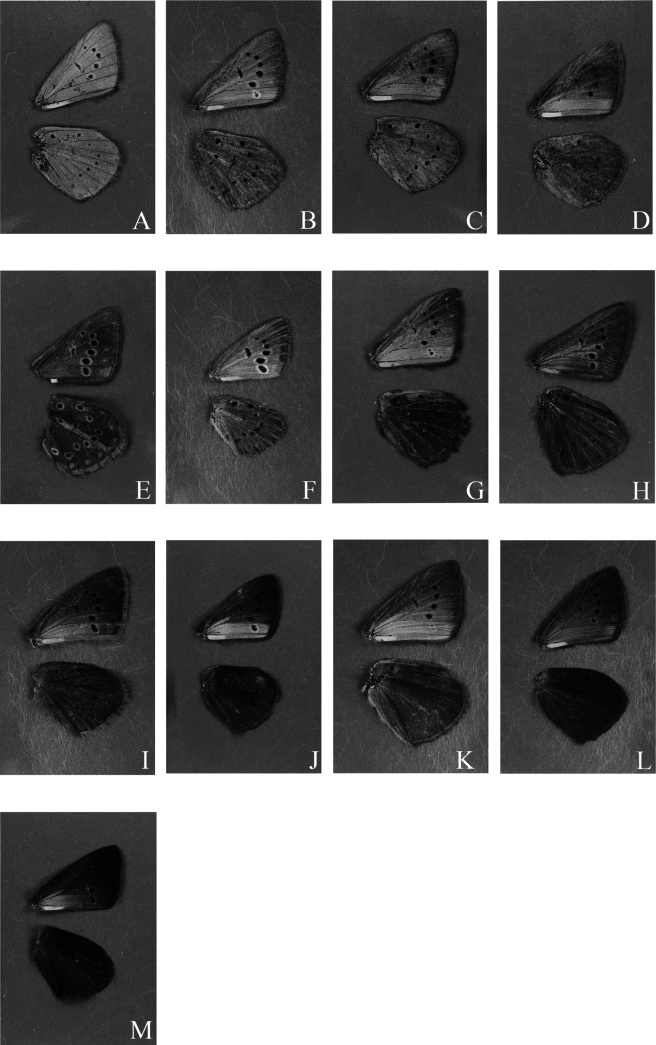
Examples of 13 different types of UV pattern on the underside of the wings. A. *P. ripartii pelopi*, D085; B. *P. pseudorjabovi*, VM723; C. *P. ripartii pelopi*, LR-08-D549; D. *P. demavendi*, VL504; E. *P. rjabovianus*, F836; F. *P. pseudorjabovi*, VM770 (female); G. *P. aroaniensis*, LR-08-D102; H. *P. valiabadi*, Z860; I. *P. admetus malievi*, VM789 (female); J. *P. khorasanensis*, F484; K. *P. demavendi belovi*, 050A07; L. *P. ripartii kalashani*, 198A08; M. *P. ripartii pelopi*, PE009 (female).

**Figure 3.**
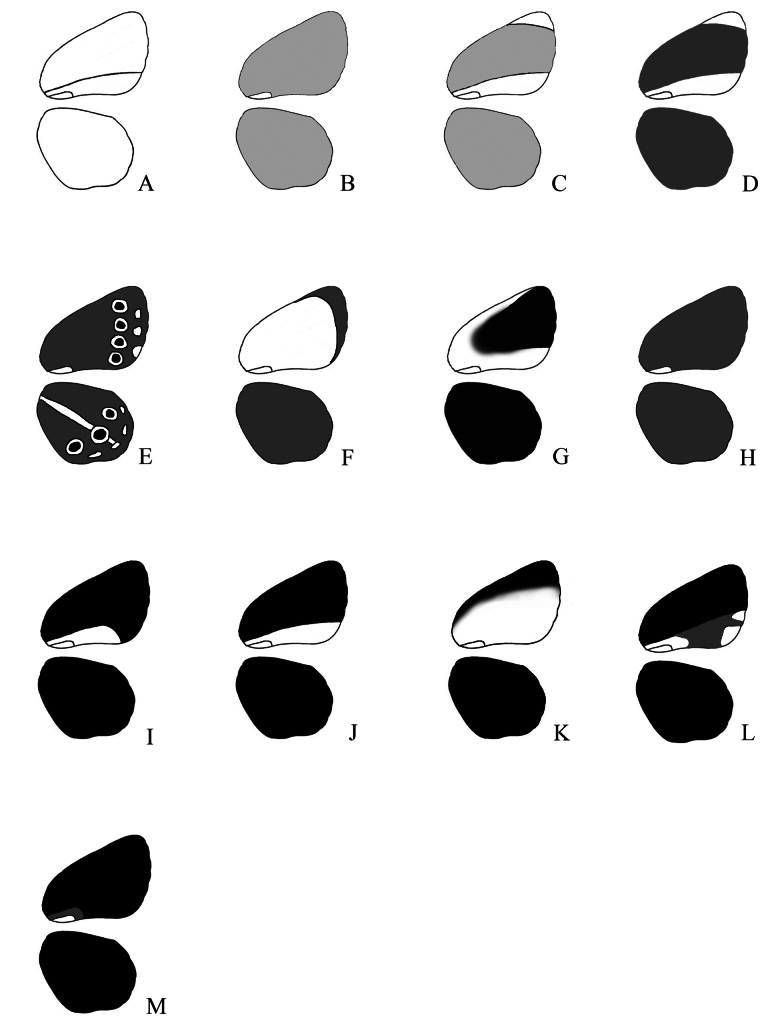
A scheme showing the 13 different UV patterns on the wing underside, based on the examples provided in Fig. 2.

We distinguished 13 different types of UV patterns (Fig. 2), which we illustrate in schemes in Fig. 3. The patterns were distinguished based on the characters described in Table 2.

**Table 2.** Character matrix of ultraviolet pattern on the underside of wings of the brown-complex species.

Characters / States	State 1	State 2	State 3	State 4	State 5
Background color of the underside of the wings	Almost white	Light grey	Grey	Dark grey, almost black	
Uniformity of background color of the underside of the wings	Uniform	With a light band of different shape along the inner margin of the underside of the forewing			
The light band along the inner margin of the underside of the forewing, it is	White and short (up to the middle of the wing) limited above by the Cu2	White and long (up to the wings margin) limited above by the Cu2	White and wide – extends above the Cu2	Grey and short (up to the middle of the wing) limited above by the Cu2	Grey and long (up to the wings margin) limited above by the Cu2, with some light marking in submarginal area
The color of the apical area of the underside of the forewing is lighter than the wing area above the Cu2	no	yes			
There is light costal area and dark area between R4+5, M3 and the Discal cell on the underside of the forewing	no	yes			
The submarginal area darker then the background of the forewing	no	yes			
The eyespots and the submarginal marking on the underside of the wing	Present but not very pronounced (not vivid)	Eyespots and the submarginal marking are very vivid, eyespots with bright white contouring			

### ﻿Description of the UV pattern types

Different combinations of the character states described in Table 2 appear in different types of UV patterns.

Type A. Background color is almost white. The background color is uniform.

Type B. Background color is light grey. The background color is uniform.

Type C. Background color is light grey. There is a long white band along the inner margin of the forewing. The band is limited above by the second cubital vein (Cu2). The apical area is lighter than the wing area above the Cu2.

Type D. Background color is grey. There is a long white band along the inner margin of the forewing. The band is limited above by the second cubital vein (Cu2). The apical area is lighter than the wing area above the Cu2. On the whole the pattern is similar to the Type C, but differs in the color of the background.

Type E. Background color is grey. The eyespots with white contouring are vivid. The submarginal marking on both wings and the white streak on the hindwing are vivid.

Type F. Background color is light grey or grey. The submarginal area is darker then the background of the forewing.

Type G. The costal area is lighter than the forewing area limited by R4+5, M3 and the Discal cell (or space 6, space 5, space 4 according to the scheme of the wing provided by Eckweiler and Bozano 2016, Lycaenidae part IV). The white band along the inner margin can be wide and extend above the Cu2.

Type H. Background color is grey. The background color is uniform.

Type I. Background color is dark grey (almost black). A white band along the inner margin of the forewing is short (it ends in the middle of the wing). The band is limited above by the Cu2.

Type J. Background color is dark grey (almost black). A white band along the inner margin of the forewing is long (it ends by the margin of the wing). The band is limited above by the Cu2.

Type K. Background color is dark grey (almost black). A white band along the inner margin of the forewing is wide: it extends above the Cu2.

Type L. Background color is dark grey (almost black). A band along the inner margin of the forewing is grey and long (it ends by the margin of the wing). The band is limited above by the Cu2. There are white (lighter) markings within the band on the margin of the forewing.

Type M. Background color is dark grey (almost black). A band along the inner margin of the forewing is grey and short (it ends in the middle of the wing).

### ﻿UV pattern distribution

The UV pattern distribution within the monomorphic complex species is demonstrated in the Table 3. It shows high variability of UV pattern within the species and between the species. The numbers in the table cells show the number of butterflies with a specific ultraviolet pattern. Though the sample size differed among species, darker types of UV pattern (ultraviolet absorption) were found in a larger proportion of butterflies. For example, the lightest pattern type A was found in four species. Interestingly, type E pattern, which was present in *P. pseudorjabovi* Lukhtanov, Dantchenko, Vishnevskaya & Saifitdinova, 2015, *P. rjabovianus* Koçak, 1980 and *P. valiabadi* Rose & Schurian, 1977, was found only in the samples, collected in 2003 and 2014. In 2024 during the expedition to the same locality (Talysh, Azerbaijan), we collected a huge sample of *P. pseudorjabovi* (63 specimens), none of which had the type E pattern.

**Table 3.** The distribution of UV pattern in monomorphic species complex.

	A	B	C	D	E	F	G	H	I	J	K	L	M
*P. admetus*		1	1	1		5	2		8	10	9	3	3
*P. alcestis*										1		1	
*P. aroaniensis*							1						
*P. dantchenkoi*								1	1	1	1		1
*P. demavendi*				5		3	6			28	13	1	
*P. emmeli*									2		1	6	1
*P. eriwanensis*												2	2
*P. keleybaricus*		2									1	1	1
*P. khorasanensis*							2			5	1	1	
*P. nephohiptamenos*				1						2		4	
*P. orphicus*							1	5			1	5	10
*P. pseudorjabovi*	8	8	3	1	2	10	4	2	4	14	4	10	1
*P. ripartii*	2	1	1				3	3	1	5	1	10	4
*P. rjabovianus*	1				5			3	1			3	3
*P. timfristos*						1					1	3	1
*P. valiabadi*		1			1			3					1
**Total number**	**11**	**13**	**5**	**8**	**8**	**19**	**19**	**17**	**17**	**54**	**33**	**50**	**28**

**Figure 4.**
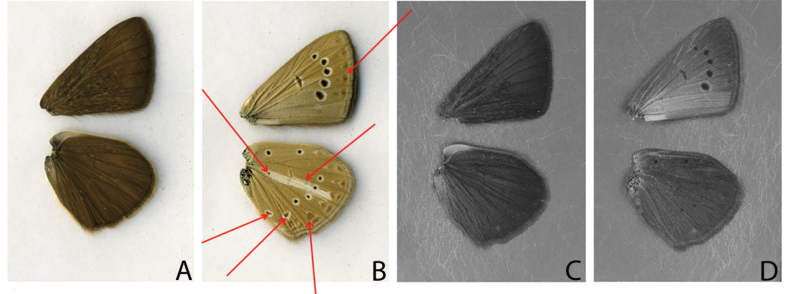
*P. admetus yeranyani* (voucher number 154A08, male) wings in visible light and UV light. A. Upperside of the wings in visible light; B. Underside of the wings in visible light; C. Upperside of the wings in UV light; D. Underside of the wings in UV light. Red arrows show the elements of the pattern in visible light which are absent in the UV light.

When we compared the patterns of the underside of the wings in visible light with those in UV light, we found that in many cases some elements of the visible pattern were not visible in the UV images (Fig. 4).

For example, Fig. 4 shows differences in the absence and presence of white streak, submarginal marking and even some black eye-spots on the underside of the wings between visible and UV spectra. In some cases, the discal spot was absent in UV light while in others the white streak was present in UV image as well as the submarginal marking and the eye-spots. As the presence or absence of pattern elements in the UV images appeared to be random between the species and within the species, we did not take them into account when we described the types of UV patterns.

### ﻿UV pattern distribution within *admetus* and *dolus* clades of the *P. admetus* lineage

**Figure 5.**
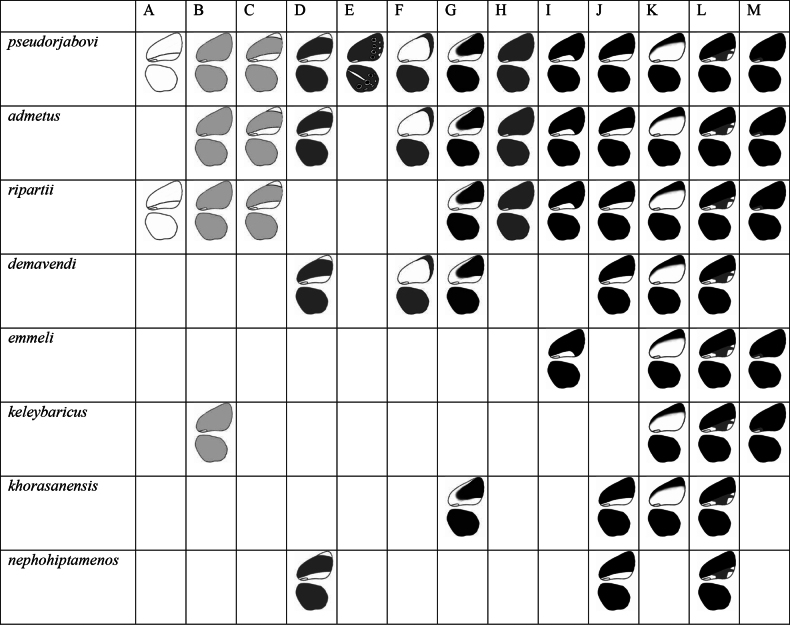
UV pattern distribution in the *admetus* group.

*Polyommatus admetus* lineage includes two sister clades: *admetus* clade and *dolus* clade, which were revealed by the analysis of two mitochondrial markers, COI and COII (see Kandul et al. 2004). Within the *admetus* group (Fig. 5) all types of UV pattern were present. For example, absolutely all types were found in *P. pseudorjabovi*. On one hand in this study *P. pseudorjabovi* was represented by the largest sample. On the other hand, the samples of *P. admetus* and *P. ripartii* (Freyer, 1830) were significantly smaller, however, they still exhibited a fairly large number of UV pattern types.

**Figure 6.**
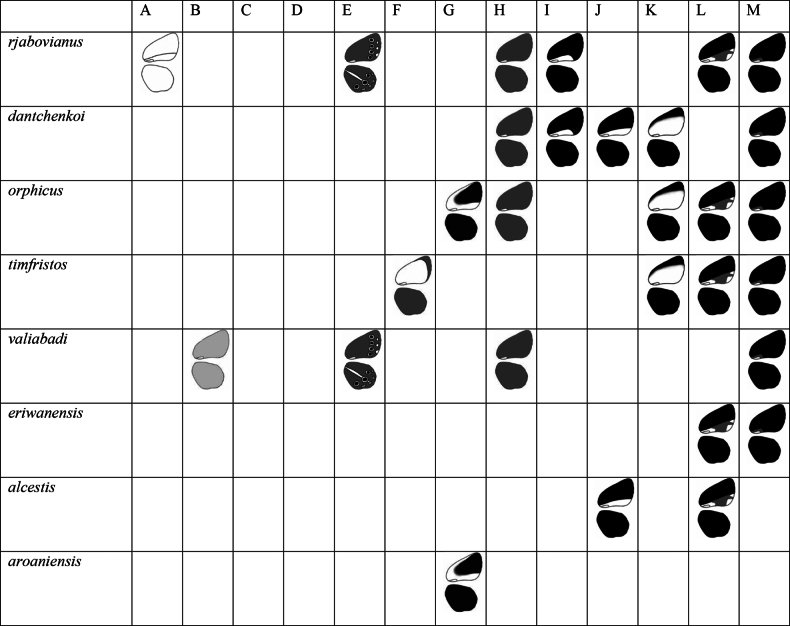
UV pattern distribution in the *dolus* group.

Within the *dolus* group, the distribution of UV pattern appeared to be quite uneven (Fig. 6). No species could demonstrate all types of UV patterns. Pattern types C and D were not present at all within the species of this group. Probably a lower sample size (in comparison to *admetus* group) was the reason for such distribution of UV pattern in the *dolus* group.

To identify patterns in the distribution of UV pattern types within the monomorphic species complex, we compared their occurrence in different species habitats, in allopatric populations of a single species, and in sympatric populations of different species.

### ﻿UV pattern analysis within males and females

Some species samples contain both sexes. UV pattern distribution between sexes is represented in Tables 4, 5. All females demonstrated dark UV pattern types but only females of *P. pseudorjabovi*, also demonstrated light pattern types D and F. Excluding females from the whole sample had little effect on the overall distribution of UV pattern types (Table 5). In *P. admetus*, pattern M was demonstrated only by females, as well as pattern D in *P. pseudorjabovi* and pattern I in *P. rjabovianus*.

**Table 4.** Distribution of UV pattern in females.

	A	B	C	D	E	F	G	H	I	J	K	L	M
*P. admetus malievi*									7	1			3
*P. orphicus orphicus*													8
*P. pseudorjabovi*				1		4	1		3	9	2	5	
*P. ripartii pelopi*													1
*P. rjabovianus rjabovianus*									1			1	

**Table 5.** Distribution of UV pattern in males of all species. In bold are the species samples which contain females, represented in the table above.

	A	B	C	D	E	F	G	H	I	J	K	L	M
***P. admetus***		1	1	1		5	2		1	9	9	3	
*P. alcestis*										1		1	
*P. aroaniensis*							1						
*P. dantchenkoi*								1	1	1	1		1
*P. demavendi*				5		3	6			28	13	1	
*P. emmeli*									2		1	6	1
*P. eriwanensis*												2	2
*P. keleybaricus*		2									1	1	1
*P. khorasanensis*							2			5	1	1	
*P. menalcas*	1						1			3	2	2	1
*P. nephohiptamenos*				1						2		4	
***P. orphicus***							1	5			1	5	2
***P. pseudorjabovi***	8	8	3		2	6	3	2	1	5	2	5	1
***P. ripartii***	2	1	1				3	3	1	5	1	10	3
***P. rjabovianus***	1				5			3				2	3
*P. timfristos*						1					1	3	1
*P. valiabadi*		1			1			3					1

### ﻿UV pattern in different localities

Within Armenian species populations we observed a shift in UV pattern to darker types with types A-H absent (Suppl. material 29a). In contrast, Azerbaijani species populations demonstrated all the variants of the UV pattern types (as it was mentioned above all patterns were documented for *P. pseudorjabovi*). Type E was found only in species *P. pseudorjabovi*, *P. rjabovianus*, and *P. valiabadi* (Suppl. material 29b). Balkan *Agrodiaetus* populations showed quite high variety of UV pattern, lacking types E and I (Suppl. material 30a). Populations of the species inhabiting Turkey did not demonstrate pattern types A-E (Suppl. material 30b). Within Iranian populations the UV pattern appeared variable, with pattern types C and I absent (Suppl. material 31a). *Agrodiaetus* butterflies in Russia were represented by five exemplars of *P. ripartii* collected in the Altai Mountains (Suppl. material 31b). These specimens demonstrated patterns H, J and K.

### ﻿UV pattern types in allopatric populations of one species

When we analyzed the distribution of patterns in allopatric populations of individual species we observed the following: in almost all cases, one common pattern was present in all allopatric populations of a species, while some patterns occurred only in populations inhabiting specific territories (see Suppl. materials 32–34). For example, in *P. demavendi* patterns J and K were common for all populations, but patterns D, F, and G were present only in Iran, and pattern L only in Armenia. In *P. orphicus*, common patterns included H, L, and M, while *P. orphicus orphicus* from Bulgaria also demonstrated patterns G and K. In *P. admetus* all populations demonstrated pattern J. In addition to a J-type pattern there were also types F and K in a population from Greece; a population from Turkey demonstrated F, G, I, K, and L; a population from Armenia type K, and a population from Azerbaijan demonstrated almost all UV pattern types except A, E, H, and L. *Polyommatus admetus* from Bulgaria and Iran displayed only the common pattern type J. In *P. rjabovianus* we observed three common types, E, H and M, while a population from Iran also demonstrated types A and L. *Polyommatus ripartii* was the only species without common pattern among its allopatric populations. However, three patterns were shared among three groups of territories: pattern J in Russia, Turkey, and Bulgaria, pattern L in Turkey, Armenia, and Greece, and pattern M in Turkey, Greece, and Bulgaria. Moreover, each of these patterns was represented by different subspecies inhabiting the respective territories.

### ﻿UV pattern types in sympatric and syntopic species

While the sample of *Agrodiaetus* species on the whole did not seem to be small, being divided into sympatric populations the number of specimens in each sample fell dramatically. Probably, this could influence the results of the analysis of UV pattern distribution in sympatric species. Nonetheless we would like to comment on the facts we observed while comparing UV pattern in sympatric populations. The distribution pattern appeared to resemble the one in allopatric populations. We observed some UV patterns which were common for several species; at the same time, we observed UV patterns which were unique to one of the species. For example, in Armenian sympatric populations in Gnyshic (Suppl. material 35a) UV patterns K and L were common for several species. At the same time species *P. demavendi* demonstrated an extra pattern J, *P. emmeli* Lukhtanov & Dantchenko, 2024 an extra pattern I, and *P. eriwanensis* Forster, 1960 an extra pattern M. In Sevan, populations of *P. emmeli* and *P. demavendi antonius* Lukhtanov & Dantchenko, 2024 did not demonstrate any common UV pattern (Suppl. material 35b).

The sample from Azerbaijan as well as the sample from Bulgaria contained females. Within Azerbaijani syntopic populations in Mistan (Suppl. material 36a) we did not observe any common pattern for *P. pseudorjabovi*, *P. admetus malievi* Dantchenko & Lukhtanov, 2004, and *P. rjabovianus*, but we observed unique patterns for populations in this area: C and D for *P. admetus malievi*, and A, B, F, and L for *P. pseudorjabovi*. In Pirasorah (Suppl. material 36b) only *P. pseudorjabovi* demonstrated unique patterns, while in Hoveri (Suppl. material 36c) each species population had specific UV pattern. In Monidigah (Suppl. material 36d), where *P. pseudorjabovi* and *P. rjabovianus* were collected, both species demonstrated common and unique patterns. When we analyzed the distribution of pattern in females, we observed a small shift to each pattern depending on a species. For example, in Mistan, *P. admetus malievi* and *P. pseudorjabovi* shared common pattern I and J; however, in *P. pseudorjabovi* pattern I was found in six individuals and pattern J in one individual, and in *P. admetus malievi* pattern J was found in six individuals and pattern I in one individual. Moreover, we also observed unique patterns: M for *P. admetus malievi* and F, K, and L for *P. pseudorjabovi*.

In Pirasorah the sample of *P. rjabovianus* was represented by the only female. At the same time *P. pseudorjabovi* demonstrated patterns which were only represented by females in this area (Suppl. material 36b).

In Monidigah both species were represented by just one exemplar of a female, but each exemplar demonstrated different UV pattern type.

In Bulgaria females of *P. ripartii pelopi* Brown, 1976 were represented by just one exemplar, while *P. orphicus* by seven exemplars. Females of both species demonstrated UV pattern type M.

On the whole in Balkan region sympatric species demonstrated more vivid differences. Thus in Greece, Timfrist (Suppl. material 37a) *P. ripartii pelopi* and *P. timfristos* Lukhtanov, Vishnevskaya & Shapoval, 2016 shared common pattern L, at the same time each population demonstrated unique patterns. In Kalavrita, three sympatric species, *P. admetus*, *P. ripartii pelopi*, and *P. aroaniensis*, demonstrated only unique patterns (Suppl. material 37b). In Granitis (Suppl. material 37c) *P. nephohiptamenos* Brown & Coutsis, 1978 and *P. orphicus eleniae* Coutsis & De Prins, 2005 demonstrated a common pattern L, as well as two unique patterns each. Finally, in Bulgaria, Hvoina, *P. orphicus orphicus* and *P. ripartii pelopi* males exhibited only unique patterns (including females both species shared common pattern M) (Suppl. material 37d).

## ﻿Discussion

Butterflies from the monomorphic species complex of the subgenus Agrodiaetus are known to be nearly identical in wing coloration and pattern in the visible spectrum. Analysis of UV patterns uncovered great variability in this respect. In total 13 different types of UV pattern are described. Moreover, we found that patterns apparent in visible light can be hidden (absent) in UV light.

First of all, we expected to see differences in the distribution of UV patterns between species, presuming that the observed variability could be connected with species divergence. However, almost all species demonstrated several types of UV patterns, the distribution of which at first glance seemed to be random. Differences between species did not correlate with divergence, and as in visible light, in the UV spectrum some patterns were common for several (or even all) species of the monomorphic complex. Both the absence of differences (interspecific and intraspecific) and the high level of polymorphism may indicate that these traits are selectively neutral. Some states are monomorphic or polymorphic and are retained because they are not under selection. At the same time, when we compared pattern distribution between two lineages – *P. admetus* and *P. dolus* – we observed that light patterns (A–F) were more common in the *admetus* group, then in the *dolus* group. We cannot say for sure whether this observation reflects a true biological difference or is simply a result of the smaller sample size of the *dolus* group.

We also expected to find correlation between UV coloration polymorphism and geography of a species, including in allopatry and sympatry, with greater differences between sympatric species, as reported previously (Lukhtanov et al. 2005). Upon closer study, we noticed some patterns in the distribution of UV coloration depending on the area of a species (Suppl. materials 29–31). Comparing patterns in different species areas we observed different variants of pattern distribution: some populations exhibited all patterns, others showed a shift toward darker pattern types, and some lacked certain patterns depending on the locality.

When analyzing pattern types in allopatric populations of individual species we observed that some patterns were shared across all populations, while others occurred only in populations from specific territories. For example, *P. demavendi* exhibited patterns J and K in all populations, but patterns D, F and G were found only in populations from Iran, while pattern L was found only in Armenian population (see Suppl. material 32a). Interestingly, *P. ripartii* did not have a single common pattern across its populations. However, three patterns were shared among three groups of territories: pattern J in Russia, Turkey, and Bulgaria; pattern L in Turkey, Armenia, and Greece; and pattern M in Turkey, Greece, and Bulgaria. Moreover, each of the patterns was represented by different subspecies inhabiting these territories

Analyzing pattern types in sympatric and syntopic populations we presumed that difference in UV pattern could occur as a result of the change of the phenotype in response to the secondary contact of the cryptic species, which appeared to live in sympatry. According to the Dobzhansky rule (Lukhtanov 2011) one of the responses to the secondary contact (an overlap of two diverged populations, which managed to accumulate a certain pool of mutations that will prevent the emergence of fertile hybrids) may be a reinforcement of prezygotic isolation, which will manifest in reinforcement of the differences between the populations and the completion of the speciation process. A study by Lukhtanov et al. in 2005 clearly demonstrated the work of this rule within dimorphic species of the subgenus Agrodiaetus (Lukhtanov et al. 2005).

Testing this hypothesis, we expected to observe the “shift” in UV pattern types in species living in sympatry, especially in syntopy. For this we compared images of the sympatric species to find out whether any type of wing pattern appeared more often. In most cases, it was hard to make conclusions since the sample size was quite small: some species were represented by fewer than ten individuals. Nonetheless, we observed instances in which each population within sympatric populations had a specific pattern unique for the area (Suppl. materials 35–37).

When we discuss the UV coloration in butterflies, we also have to bear in mind the fact that one of the factors affecting the expression of UV patterns can be the larval food plant. Some butterflies from different families, including Papilionidae, Nymphalidae and Lycaenidae, can sequester flavonoids from their larval host plants (Wilson 1986, 1987; Nijhout 1991, Wiesen et al. 1994; Burghardt et al. 1997; Schittko et al. 1999). For example, Burghardt and coauthors in their study performed series of experiments where caterpillars of *P. icarus* (Rottemburg, 1775) fed on different parts of the same plant – flavonoid-rich inflorescence or flavonoid-poor foliage – which resulted in imago females having different UV patterns on their wings (Burghardt et al, 2000). Moreover, *P. icarus* is polyphagous, so UV pattern strongly depends on which part or which host plant the caterpillar was feeding.

There is no data on *Agrodiaetus* butterflies’ ability to sequester flavonoids from their larval host plants, and this study did not allow us to test it, but we cannot reject this idea. *Agrodiaetus* butterflies are known to be monophagous. If we presume that *Agrodiaetus* caterpillars also sequester flavonoids, then we can interpret the intensity of UV reflection and absorption by the preference of flavonoid-rich inflorescence or flavonoid-poor foliage by the caterpillar. We could also explain the shift to darker UV patterns in females, since for *P. icarus* it is experimentally proved that females sequester more flavonoids and usually absorb UV light (Burghardt et al. 2000; Knüttel and Fiedler 2001).

On the other hand, if we presume that *Agrodiaetus* caterpillars also sequester flavonoids, we could ask a different question: are these butterflies monophagous?

In light of the fact that butterflies of the subgenus Agrodiaetus demonstrate the presence of unique color patterns in the UV spectrum in allopatry (and sympatry), as well as the fact that some species of the genus *Polyommatus* demonstrate the dependence of wing color in the UV spectrum on the food supply of the caterpillar, gives us the opportunity to assume that, in theory, differences in UV pattern may be associated with the transition of caterpillars to different plants in allopatry. This also could explain the shift in UV pattern to darker types or the appearance of light types in different geographical areas. We cannot yet prove this experimentally, but we can put forward such a hypothesis, that *Agrodiaetus* butterflies could be oligophagous, or change their food specialization (move from one plant species to another) depending on the area, which is reflected in the UV pattern in representatives of allopatric populations of the same species or variety in UV pattern in different geographical area.

Concerning *Agrodiaetus* species with high variability in UV patterns living in syntopy, we presume that caterpillars can compete for inflorescence, which results in higher proportion of specimens with darker UV pattern types compared to lighter ones. Previous work (Burghardt et al. 2001; Knüttel and Fiedler 2001) based on experiments with the common blue butterfly *P. icarus* show that females at larval stages which fed on parts of the plant rich in flavonoids (inflorescences), were larger in size and darker in UV (they absorb UV), which was more attractive to males. This suggests that caterpillars feeding on inflorescence have a higher reproductive success in the imago stage than those feeding on foliage. In theory, the differentiation of caterpillars into two groups (feeding on foliage and feeding on inflorescence) could explain the presence of conspecifics with different levels of UV reflection and absorption, living in sympatry. Some authors doubt that UV patterns can serve in species recognition (Knüttel and Fiedler 2000; Burghardt et al. 2000) since it depends on the larval host plant. This issue remains controversial. The choice of a “wrong” part of the host plant by the caterpillar may lead to elimination of the imago individuals which will have a “wrong” UV pattern. Maybe this can explain the “shift” to the darker types of UV patterns in *Agrodiaetus* species not only in females, but also in males.

## ﻿Conclusions

The high variability in UV coloration in butterflies of the monomorphic species complex discovered in this study, as well as description of 13 UV pattern types did not give a clear answer to the question on how the studied cryptic species recognize conspecifics. However, we revealed some pattern in the distribution of UV coloration depending upon the habitat of the species. Of course, to draw more accurate conclusions about the correlation between the UV pattern and various factors that could influence it, a significantly larger sample size and years of observations are required. We cannot use the UV to distinguish between the species, and still cannot answer the question on intra- or interspecific recognition within cryptic species, but we can put forward hypotheses whether the difference in UV pattern depends on the larval host plant within the monomorphic species complex of the subgenus Agrodiaetus.

## ﻿Additional information

### Use of AI

No use of AI was reported.

### Author ORCIDs

Maria S. Vishnevskaya https://orcid.org/0000-0002-2634-2610

Vladimir A. Lukhtanov https://orcid.org/0000-0003-2856-2075

### Data availability

All of the data that support the findings of this study are available in the main text or Supplementary Information.

## Supplementary materials

Supplementary material 1 List of studied species Data type xlsx Explanation note Includes information on species names, voucher numbers, GenBank ID, chromosome numbers, UV pattern types, localities, date. Supplied with references on publications for previously studied species. https://binary.pensoft.net/file/1444543This dataset is made available under the Open Database License (http://opendatacommons.org/licenses/odbl/1.0/). The Open Database License (ODbL) is a license agreement intended to allow users to freely share, modify, and use this Dataset while maintaining this same freedom for others, provided that the original source and author(s) are credited.Maria S. Vishnevskaya, Vladimir A. Lukhtanov, Alexander V. Dantchenko, Elizaveta Yu. Gorodilova

Supplementary material 2 Specimens of *P. admetus* in visible light and UV light, part 1 Data type tif Explanation note Numbers under the photos indicate field voucher numbers of each specimen. First two images of each specimen are photos of upperside and underside of the wings in visible light, next two images – photos of upperside and underside of the wings in UV light. Scale bar: 1 cm. https://binary.pensoft.net/file/1444544This dataset is made available under the Open Database License (http://opendatacommons.org/licenses/odbl/1.0/). The Open Database License (ODbL) is a license agreement intended to allow users to freely share, modify, and use this Dataset while maintaining this same freedom for others, provided that the original source and author(s) are credited.Maria S. Vishnevskaya, Vladimir A. Lukhtanov, Alexander V. Dantchenko, Elizaveta Yu. Gorodilova

Supplementary material 3 Specimens of *P. admetus* in visible light and UV light, part 2 Data type tif Explanation note Numbers under the photos indicate field voucher numbers of each specimen. First two images of each specimen are photos of upperside and underside of the wings in visible light, next two images – photos of upperside and underside of the wings in UV light. Scale bar: 1 cm. https://binary.pensoft.net/file/1444545This dataset is made available under the Open Database License (http://opendatacommons.org/licenses/odbl/1.0/). The Open Database License (ODbL) is a license agreement intended to allow users to freely share, modify, and use this Dataset while maintaining this same freedom for others, provided that the original source and author(s) are credited.Maria S. Vishnevskaya, Vladimir A. Lukhtanov, Alexander V. Dantchenko, Elizaveta Yu. Gorodilova

Supplementary material 4 Specimens of *P. admetus* in visible light and UV light, part 3 Data type tif Explanation note Numbers under the photos indicate field voucher numbers of each specimen. First two images of each specimen are photos of upperside and underside of the wings in visible light, next two images – photos of upperside and underside of the wings in UV light. Scale bar: 1 cm. https://binary.pensoft.net/file/1444546This dataset is made available under the Open Database License (http://opendatacommons.org/licenses/odbl/1.0/). The Open Database License (ODbL) is a license agreement intended to allow users to freely share, modify, and use this Dataset while maintaining this same freedom for others, provided that the original source and author(s) are credited.Maria S. Vishnevskaya, Vladimir A. Lukhtanov, Alexander V. Dantchenko, Elizaveta Yu. Gorodilova

Supplementary material 5 Specimens of *P. admetus* (part 4), *P. aroaniensis*, *P. alcestis* in visible light and UV light Data type tif Explanation note Numbers under the photos indicate field voucher numbers of each specimen: *P. admetus* – VM788, VM789, VM790, VM796, VM626, VM674, VM802, *P. aroaniensis* – LR-08-D102, *P. alcestis* – N504, N512. First two images of each specimen are photos of upperside and underside of the wings in visible light, next two images – photos of upperside and underside of the wings in UV light. Scale bar: 1 cm. https://binary.pensoft.net/file/1444547This dataset is made available under the Open Database License (http://opendatacommons.org/licenses/odbl/1.0/). The Open Database License (ODbL) is a license agreement intended to allow users to freely share, modify, and use this Dataset while maintaining this same freedom for others, provided that the original source and author(s) are credited.Maria S. Vishnevskaya, Vladimir A. Lukhtanov, Alexander V. Dantchenko, Elizaveta Yu. Gorodilova

Supplementary material 6 Specimens of *P. demavendi* in visible light and UV light, part 1 Data type tif Explanation note Numbers under the photos indicate field voucher numbers of each specimen. First two images of each specimen are photos of upperside and underside of the wings in visible light, next two images – photos of upperside and underside of the wings in UV light. Scale bar: 1 cm. https://binary.pensoft.net/file/1444548This dataset is made available under the Open Database License (http://opendatacommons.org/licenses/odbl/1.0/). The Open Database License (ODbL) is a license agreement intended to allow users to freely share, modify, and use this Dataset while maintaining this same freedom for others, provided that the original source and author(s) are credited.Maria S. Vishnevskaya, Vladimir A. Lukhtanov, Alexander V. Dantchenko, Elizaveta Yu. Gorodilova

Supplementary material 7 Specimens of *P. demavendi* in visible light and UV light, part 2 Data type tif Explanation note Numbers under the photos indicate field voucher numbers of each specimen. First two images of each specimen are photos of upperside and underside of the wings in visible light, next two images – photos of upperside and underside of the wings in UV light. Scale bar: 1 cm. https://binary.pensoft.net/file/1444549This dataset is made available under the Open Database License (http://opendatacommons.org/licenses/odbl/1.0/). The Open Database License (ODbL) is a license agreement intended to allow users to freely share, modify, and use this Dataset while maintaining this same freedom for others, provided that the original source and author(s) are credited.Maria S. Vishnevskaya, Vladimir A. Lukhtanov, Alexander V. Dantchenko, Elizaveta Yu. Gorodilova

Supplementary material 8 Specimens of *P. demavendi* in visible light and UV light, part 3 Data type tif Explanation note Numbers under the photos indicate field voucher numbers of each specimen. First two images of each specimen are photos of upperside and underside of the wings in visible light, next two images – photos of upperside and underside of the wings in UV light. Scale bar: 1 cm. https://binary.pensoft.net/file/1444550This dataset is made available under the Open Database License (http://opendatacommons.org/licenses/odbl/1.0/). The Open Database License (ODbL) is a license agreement intended to allow users to freely share, modify, and use this Dataset while maintaining this same freedom for others, provided that the original source and author(s) are credited.Maria S. Vishnevskaya, Vladimir A. Lukhtanov, Alexander V. Dantchenko, Elizaveta Yu. Gorodilova

Supplementary material 9 Specimens of *P. demavendi lorestanus* in visible light and UV light, part 1 Data type tif Explanation note Numbers under the photos indicate field voucher numbers of each specimen. First two images of each specimen are photos of upperside and underside of the wings in visible light, next two images – photos of upperside and underside of the wings in UV light. Scale bar: 1 cm. https://binary.pensoft.net/file/1444551This dataset is made available under the Open Database License (http://opendatacommons.org/licenses/odbl/1.0/). The Open Database License (ODbL) is a license agreement intended to allow users to freely share, modify, and use this Dataset while maintaining this same freedom for others, provided that the original source and author(s) are credited.Maria S. Vishnevskaya, Vladimir A. Lukhtanov, Alexander V. Dantchenko, Elizaveta Yu. Gorodilova

Supplementary material 10 Specimens of *P. demavendi lorestanus* in visible light and UV light, part 2 Data type tif Explanation note Numbers under the photos indicate field voucher numbers of each specimen. First two images of each specimen are photos of upperside and underside of the wings in visible light, next two images – photos of upperside and underside of the wings in UV light. Scale bar: 1 cm. https://binary.pensoft.net/file/1444552This dataset is made available under the Open Database License (http://opendatacommons.org/licenses/odbl/1.0/). The Open Database License (ODbL) is a license agreement intended to allow users to freely share, modify, and use this Dataset while maintaining this same freedom for others, provided that the original source and author(s) are credited.Maria S. Vishnevskaya, Vladimir A. Lukhtanov, Alexander V. Dantchenko, Elizaveta Yu. Gorodilova

Supplementary material 11 Specimens of *P. emmeli* in visible light and UV light Data type tif Explanation note Numbers under the photos indicate field voucher numbers of each specimen. First two images of each specimen are photos of upperside and underside of the wings in visible light, next two images – photos of upperside and underside of the wings in UV light. Scale bar: 1 cm. https://binary.pensoft.net/file/1444553This dataset is made available under the Open Database License (http://opendatacommons.org/licenses/odbl/1.0/). The Open Database License (ODbL) is a license agreement intended to allow users to freely share, modify, and use this Dataset while maintaining this same freedom for others, provided that the original source and author(s) are credited.Maria S. Vishnevskaya, Vladimir A. Lukhtanov, Alexander V. Dantchenko, Elizaveta Yu. Gorodilova

Supplementary material 12 Specimens of *P. eriwanensis* and *P. dantchenkoi* in visible light and UV light Data type tif Explanation note Numbers under the photos indicate field voucher numbers of each specimen: *P. eriwanensis* – 007A07, 008A07, 019A07, 020A07, *P. dantchenkoi* – L341–L345. First two images of each specimen are photos of upperside and underside of the wings in visible light, next two images – photos of upperside and underside of the wings in UV light. Scale bar: 1 cm. https://binary.pensoft.net/file/1444554This dataset is made available under the Open Database License (http://opendatacommons.org/licenses/odbl/1.0/). The Open Database License (ODbL) is a license agreement intended to allow users to freely share, modify, and use this Dataset while maintaining this same freedom for others, provided that the original source and author(s) are credited.Maria S. Vishnevskaya, Vladimir A. Lukhtanov, Alexander V. Dantchenko, Elizaveta Yu. Gorodilova

Supplementary material 13 Specimens of *P. khorasanensis* in visible light and UV light Data type tif Explanation note Numbers under the photos indicate field voucher numbers of each specimen. First two images of each specimen are photos of upperside and underside of the wings in visible light, next two images – photos of upperside and underside of the wings in UV light. Scale bar: 1 cm. https://binary.pensoft.net/file/1444555This dataset is made available under the Open Database License (http://opendatacommons.org/licenses/odbl/1.0/). The Open Database License (ODbL) is a license agreement intended to allow users to freely share, modify, and use this Dataset while maintaining this same freedom for others, provided that the original source and author(s) are credited.Maria S. Vishnevskaya, Vladimir A. Lukhtanov, Alexander V. Dantchenko, Elizaveta Yu. Gorodilova

Supplementary material 14 Specimens of *P. nephohiptamenos* in visible light and UV light Data type tif Explanation note Numbers under the photos indicate field voucher numbers of each specimen. First two images of each specimen are photos of upperside and underside of the wings in visible light, next two images – photos of upperside and underside of the wings in UV light. Scale bar: 1 cm. https://binary.pensoft.net/file/1444556This dataset is made available under the Open Database License (http://opendatacommons.org/licenses/odbl/1.0/). The Open Database License (ODbL) is a license agreement intended to allow users to freely share, modify, and use this Dataset while maintaining this same freedom for others, provided that the original source and author(s) are credited.Maria S. Vishnevskaya, Vladimir A. Lukhtanov, Alexander V. Dantchenko, Elizaveta Yu. Gorodilova

Supplementary material 15 Specimens of *P. orphicus* in visible light and UV light, part 1 Data type tif Explanation note Numbers under the photos indicate field voucher numbers of each specimen. First two images of each specimen are photos of upperside and underside of the wings in visible light, next two images – photos of upperside and underside of the wings in UV light. Scale bar: 1 cm. https://binary.pensoft.net/file/1444557This dataset is made available under the Open Database License (http://opendatacommons.org/licenses/odbl/1.0/). The Open Database License (ODbL) is a license agreement intended to allow users to freely share, modify, and use this Dataset while maintaining this same freedom for others, provided that the original source and author(s) are credited.Maria S. Vishnevskaya, Vladimir A. Lukhtanov, Alexander V. Dantchenko, Elizaveta Yu. Gorodilova

Supplementary material 16 Specimens of *P. orphicus* in visible light and UV light, part 2 Data type tif Explanation note Numbers under the photos indicate field voucher numbers of each specimen. First two images of each specimen are photos of upperside and underside of the wings in visible light, next two images – photos of upperside and underside of the wings in UV light. Scale bar: 1 cm. https://binary.pensoft.net/file/1444558This dataset is made available under the Open Database License (http://opendatacommons.org/licenses/odbl/1.0/). The Open Database License (ODbL) is a license agreement intended to allow users to freely share, modify, and use this Dataset while maintaining this same freedom for others, provided that the original source and author(s) are credited.Maria S. Vishnevskaya, Vladimir A. Lukhtanov, Alexander V. Dantchenko, Elizaveta Yu. Gorodilova

Supplementary material 17 Specimens of *P. pseudorjabovi* in visible light and UV light, part 1 Data type tif Explanation note Numbers under the photos indicate field voucher numbers of each specimen. First two images of each specimen are photos of upperside and underside of the wings in visible light, next two images – photos of upperside and underside of the wings in UV light. Scale bar: 1 cm. https://binary.pensoft.net/file/1444559This dataset is made available under the Open Database License (http://opendatacommons.org/licenses/odbl/1.0/). The Open Database License (ODbL) is a license agreement intended to allow users to freely share, modify, and use this Dataset while maintaining this same freedom for others, provided that the original source and author(s) are credited.Maria S. Vishnevskaya, Vladimir A. Lukhtanov, Alexander V. Dantchenko, Elizaveta Yu. Gorodilova

Supplementary material 18 Specimens of *P. pseudorjabovi* in visible light and UV light, part 2 Data type tif Explanation note Numbers under the photos indicate field voucher numbers of each specimen. First two images of each specimen are photos of upperside and underside of the wings in visible light, next two images – photos of upperside and underside of the wings in UV light. Scale bar: 1 cm. https://binary.pensoft.net/file/1444560This dataset is made available under the Open Database License (http://opendatacommons.org/licenses/odbl/1.0/). The Open Database License (ODbL) is a license agreement intended to allow users to freely share, modify, and use this Dataset while maintaining this same freedom for others, provided that the original source and author(s) are credited.Maria S. Vishnevskaya, Vladimir A. Lukhtanov, Alexander V. Dantchenko, Elizaveta Yu. Gorodilova

Supplementary material 19 Specimens of *P. pseudorjabovi* in visible light and UV light, part 3 Data type tif Explanation note Numbers under the photos indicate field voucher numbers of each specimen. First two images of each specimen are photos of upperside and underside of the wings in visible light, next two images – photos of upperside and underside of the wings in UV light. Scale bar: 1 cm. https://binary.pensoft.net/file/1444561This dataset is made available under the Open Database License (http://opendatacommons.org/licenses/odbl/1.0/). The Open Database License (ODbL) is a license agreement intended to allow users to freely share, modify, and use this Dataset while maintaining this same freedom for others, provided that the original source and author(s) are credited.Maria S. Vishnevskaya, Vladimir A. Lukhtanov, Alexander V. Dantchenko, Elizaveta Yu. Gorodilova

Supplementary material 20 Specimens of *P. pseudorjabovi* in visible light and UV light, part 4 Data type tif Explanation note Numbers under the photos indicate field voucher numbers of each specimen. First two images of each specimen are photos of upperside and underside of the wings in visible light, next two images – photos of upperside and underside of the wings in UV light. Scale bar: 1 cm. https://binary.pensoft.net/file/1444562This dataset is made available under the Open Database License (http://opendatacommons.org/licenses/odbl/1.0/). The Open Database License (ODbL) is a license agreement intended to allow users to freely share, modify, and use this Dataset while maintaining this same freedom for others, provided that the original source and author(s) are credited.Maria S. Vishnevskaya, Vladimir A. Lukhtanov, Alexander V. Dantchenko, Elizaveta Yu. Gorodilova

Supplementary material 21 Specimens of *P. pseudorjabovi* in visible light and UV light, part 5 Data type tif Explanation note Numbers under the photos indicate field voucher numbers of each specimen. First two images of each specimen are photos of upperside and underside of the wings in visible light, next two images – photos of upperside and underside of the wings in UV light. Scale bar: 1 cm. https://binary.pensoft.net/file/1444563This dataset is made available under the Open Database License (http://opendatacommons.org/licenses/odbl/1.0/). The Open Database License (ODbL) is a license agreement intended to allow users to freely share, modify, and use this Dataset while maintaining this same freedom for others, provided that the original source and author(s) are credited.Maria S. Vishnevskaya, Vladimir A. Lukhtanov, Alexander V. Dantchenko, Elizaveta Yu. Gorodilova

Supplementary material 22 Specimens of *P. pseudorjabovi* in visible light and UV light, part 6 Data type tif Explanation note Numbers under the photos indicate field voucher numbers of each specimen. First two images of each specimen are photos of upperside and underside of the wings in visible light, next two images – photos of upperside and underside of the wings in UV light. Scale bar: 1 cm. https://binary.pensoft.net/file/1444564This dataset is made available under the Open Database License (http://opendatacommons.org/licenses/odbl/1.0/). The Open Database License (ODbL) is a license agreement intended to allow users to freely share, modify, and use this Dataset while maintaining this same freedom for others, provided that the original source and author(s) are credited.Maria S. Vishnevskaya, Vladimir A. Lukhtanov, Alexander V. Dantchenko, Elizaveta Yu. Gorodilova

Supplementary material 23 Specimens of *P. pseudorjabovi* (part 7) and *P. rjabovianus* (part 1) in visible light and UV light Data type tif Explanation note Numbers under the photos indicate field voucher numbers of each specimen: *P. pseudorjabovi* – VM794, VM797, VM798, *P. rjabovianus* – VM591, VM696, VM697, VM772, VL474–VL478. First two images of each specimen are photos of upperside and underside of the wings in visible light, next two images – photos of upperside and underside of the wings in UV light. Scale bar: 1 cm. https://binary.pensoft.net/file/1444565This dataset is made available under the Open Database License (http://opendatacommons.org/licenses/odbl/1.0/). The Open Database License (ODbL) is a license agreement intended to allow users to freely share, modify, and use this Dataset while maintaining this same freedom for others, provided that the original source and author(s) are credited.Maria S. Vishnevskaya, Vladimir A. Lukhtanov, Alexander V. Dantchenko, Elizaveta Yu. Gorodilova

Supplementary material 24 Specimens of *P. rjabovianus* in visible light and UV light, part 2 Data type tif Explanation note Numbers under the photos indicate field voucher numbers of each specimen. First two images of each specimen are photos of upperside and underside of the wings in visible light, next two images – photos of upperside and underside of the wings in UV light. Scale bar: 1 cm. https://binary.pensoft.net/file/1444566This dataset is made available under the Open Database License (http://opendatacommons.org/licenses/odbl/1.0/). The Open Database License (ODbL) is a license agreement intended to allow users to freely share, modify, and use this Dataset while maintaining this same freedom for others, provided that the original source and author(s) are credited.Maria S. Vishnevskaya, Vladimir A. Lukhtanov, Alexander V. Dantchenko, Elizaveta Yu. Gorodilova

Supplementary material 25 Specimens of *P. ripartii*, in visible light and UV light, part 1 Data type tif Explanation note Numbers under the photos indicate field voucher numbers of each specimen. First two images of each specimen are photos of upperside and underside of the wings in visible light, next two images – photos of upperside and underside of the wings in UV light. Scale bar: 1 cm. https://binary.pensoft.net/file/1444567This dataset is made available under the Open Database License (http://opendatacommons.org/licenses/odbl/1.0/). The Open Database License (ODbL) is a license agreement intended to allow users to freely share, modify, and use this Dataset while maintaining this same freedom for others, provided that the original source and author(s) are credited.Maria S. Vishnevskaya, Vladimir A. Lukhtanov, Alexander V. Dantchenko, Elizaveta Yu. Gorodilova

Supplementary material 26 Specimens of *P. ripartii*, in visible light and UV light, part 2 Data type tif Explanation note Numbers under the photos indicate field voucher numbers of each specimen. First two images of each specimen are photos of upperside and underside of the wings in visible light, next two images – photos of upperside and underside of the wings in UV light. Scale bar: 1 cm. https://binary.pensoft.net/file/1444568This dataset is made available under the Open Database License (http://opendatacommons.org/licenses/odbl/1.0/). The Open Database License (ODbL) is a license agreement intended to allow users to freely share, modify, and use this Dataset while maintaining this same freedom for others, provided that the original source and author(s) are credited.Maria S. Vishnevskaya, Vladimir A. Lukhtanov, Alexander V. Dantchenko, Elizaveta Yu. Gorodilova

Supplementary material 27 Specimens of *P. ripartii* (part 3) and *P. keleybaricus* in visible light and UV light Data type tif Explanation note Numbers under the photos indicate field voucher numbers of each specimen: *P. ripartii* – L106, L165, L166, L177, L464, L465, *P. keleybaricus* – L466, E250, E258 – E262. First two images of each specimen are photos of upperside and underside of the wings in visible light, next two images – photos of upperside and underside of the wings in UV light. Scale bar: 1 cm. https://binary.pensoft.net/file/1444569This dataset is made available under the Open Database License (http://opendatacommons.org/licenses/odbl/1.0/). The Open Database License (ODbL) is a license agreement intended to allow users to freely share, modify, and use this Dataset while maintaining this same freedom for others, provided that the original source and author(s) are credited.Maria S. Vishnevskaya, Vladimir A. Lukhtanov, Alexander V. Dantchenko, Elizaveta Yu. Gorodilova

Supplementary material 28 Specimens of *P. timfristos* and *P. valiabadi* in visible light and UV light Data type tif Explanation note Numbers under the photos indicate field voucher numbers of each specimen: *P. timfristos* – LR-08-D205, D247, D255, D258, D273, D274, *P. valiabadi* – Z860–Z865. First two images of each specimen are photos of upperside and underside of the wings in visible light, next two images – photos of upperside and underside of the wings in UV light. Scale bar: 1 cm. https://binary.pensoft.net/file/1444570This dataset is made available under the Open Database License (http://opendatacommons.org/licenses/odbl/1.0/). The Open Database License (ODbL) is a license agreement intended to allow users to freely share, modify, and use this Dataset while maintaining this same freedom for others, provided that the original source and author(s) are credited.Maria S. Vishnevskaya, Vladimir A. Lukhtanov, Alexander V. Dantchenko, Elizaveta Yu. Gorodilova

Supplementary material 29 UV pattern analysis. Distribution of UV pattern types in species, inhabiting Armenia and Azerbaijan Data type tif Explanation note Distribution of UV pattern types in species: **a.** Inhabiting Armenia; **b.** Inhabiting Azerbaijan. Schematic interpretation. https://binary.pensoft.net/file/1444571This dataset is made available under the Open Database License (http://opendatacommons.org/licenses/odbl/1.0/). The Open Database License (ODbL) is a license agreement intended to allow users to freely share, modify, and use this Dataset while maintaining this same freedom for others, provided that the original source and author(s) are credited.Maria S. Vishnevskaya, Vladimir A. Lukhtanov, Alexander V. Dantchenko, Elizaveta Yu. Gorodilova

Supplementary material 30 UV pattern analysis. Distribution of UV pattern types in species, inhabiting Balkan Peninsula and Turkey Data type tif Explanation note Distribution of UV pattern types in species: **a.** Inhabiting Balkan Peninsula; **b.** Inhabiting Turkey. Schematic interpretation. https://binary.pensoft.net/file/1444572This dataset is made available under the Open Database License (http://opendatacommons.org/licenses/odbl/1.0/). The Open Database License (ODbL) is a license agreement intended to allow users to freely share, modify, and use this Dataset while maintaining this same freedom for others, provided that the original source and author(s) are credited.Maria S. Vishnevskaya, Vladimir A. Lukhtanov, Alexander V. Dantchenko, Elizaveta Yu. Gorodilova

Supplementary material 31 UV pattern analysis. Distribution of UV pattern types in species, inhabiting Iran and Russia Data type tif Explanation note Distribution of UV pattern in species: **a.** Inhabiting Iran; **b.** Inhabiting Russia. Schematic interpretation. https://binary.pensoft.net/file/1444573This dataset is made available under the Open Database License (http://opendatacommons.org/licenses/odbl/1.0/). The Open Database License (ODbL) is a license agreement intended to allow users to freely share, modify, and use this Dataset while maintaining this same freedom for others, provided that the original source and author(s) are credited.Maria S. Vishnevskaya, Vladimir A. Lukhtanov, Alexander V. Dantchenko, Elizaveta Yu. Gorodilova

Supplementary material 32 UV pattern types in allopatric populations of one species: *P. demavendi*, *P. orphicus*, *P. rjabovianus* Data type tif Explanation note Distribution of UV pattern in allopatric populations of: **a.***P. demavendi*; **b.***P. orphicus*; **c.***P. rjabovianus*. Schematic interpretation. https://binary.pensoft.net/file/1444574This dataset is made available under the Open Database License (http://opendatacommons.org/licenses/odbl/1.0/). The Open Database License (ODbL) is a license agreement intended to allow users to freely share, modify, and use this Dataset while maintaining this same freedom for others, provided that the original source and author(s) are credited.Maria S. Vishnevskaya, Vladimir A. Lukhtanov, Alexander V. Dantchenko, Elizaveta Yu. Gorodilova

Supplementary material 33 UV pattern types in allopatric populations of one species: *P. ripartii* Data type tif Explanation note Distribution of UV pattern in allopatric populations of *P. ripartii*. Schematic interpretation. https://binary.pensoft.net/file/1444575This dataset is made available under the Open Database License (http://opendatacommons.org/licenses/odbl/1.0/). The Open Database License (ODbL) is a license agreement intended to allow users to freely share, modify, and use this Dataset while maintaining this same freedom for others, provided that the original source and author(s) are credited.Maria S. Vishnevskaya, Vladimir A. Lukhtanov, Alexander V. Dantchenko, Elizaveta Yu. Gorodilova

Supplementary material 34 UV pattern types in allopatric populations of one species: *P. admetus* Data type tif Explanation note Distribution of UV pattern in allopatric populations of *P. admetus*. Schematic interpretation. https://binary.pensoft.net/file/1444577This dataset is made available under the Open Database License (http://opendatacommons.org/licenses/odbl/1.0/). The Open Database License (ODbL) is a license agreement intended to allow users to freely share, modify, and use this Dataset while maintaining this same freedom for others, provided that the original source and author(s) are credited.Maria S. Vishnevskaya, Vladimir A. Lukhtanov, Alexander V. Dantchenko, Elizaveta Yu. Gorodilova

Supplementary material 35 UV pattern types in sympatric species in Armenia Data type tif Explanation note Distribution of UV pattern types in sympatric species in Armenia: **a.**Gnyshic; **b.** Sevan. Numbers in cells represent the number of specimens of each pattern-type of the species. https://binary.pensoft.net/file/1444578This dataset is made available under the Open Database License (http://opendatacommons.org/licenses/odbl/1.0/). The Open Database License (ODbL) is a license agreement intended to allow users to freely share, modify, and use this Dataset while maintaining this same freedom for others, provided that the original source and author(s) are credited.Maria S. Vishnevskaya, Vladimir A. Lukhtanov, Alexander V. Dantchenko, Elizaveta Yu. Gorodilova

Supplementary material 36 UV pattern types in sympatric species in Azerbaijan Data type tif Explanation note Distribution of UV pattern in syntopic species in Azerbaijan (orange color indicates UV pattern which is represented only by females, green color indicates UV pattern which is represented by both sexes): **a.** Mistan (*P. admetus malievi*: pattern I – 6 females, pattern J – 1 female; *P. pseudorjabovi* – pattern F – 2 females, pattern I – 1 female, pattern J – 6 females, pattern K – 1 female, pattern L – 3 females); **b.** Pirasorah (*P. pseudorjabovi*: pattern F – 2 females); **c.** Hoveri; **d.** Monidigah (*P. pseudorjabovi*: pattern J – 1 female). Numbers in cells represent the number of specimens of each pattern-type of the species. Schematic interpretation. https://binary.pensoft.net/file/1444579This dataset is made available under the Open Database License (http://opendatacommons.org/licenses/odbl/1.0/). The Open Database License (ODbL) is a license agreement intended to allow users to freely share, modify, and use this Dataset while maintaining this same freedom for others, provided that the original source and author(s) are credited.Maria S. Vishnevskaya, Vladimir A. Lukhtanov, Alexander V. Dantchenko, Elizaveta Yu. Gorodilova

Supplementary material 37 UV pattern types in sympatric species in Balkan Peninsula Data type tif Explanation note Distribution of UV pattern in sympatric species in Balkan Peninsula (orange color indicates UV pattern which is represented only by females, green color indicates UV pattern which is represented by both sexes): **a.** Greece, Timfrist; **b.** Greece, Kalavrita; **c.** Greece, Granitis; **d.** Bulgaria, Hvoina (*P. orphicus*: pattern M – 7 females). Numbers in cells represent the number of specimens of each pattern-type of the species. Schematic interpretation. https://binary.pensoft.net/file/1444580This dataset is made available under the Open Database License (http://opendatacommons.org/licenses/odbl/1.0/). The Open Database License (ODbL) is a license agreement intended to allow users to freely share, modify, and use this Dataset while maintaining this same freedom for others, provided that the original source and author(s) are credited.Maria S. Vishnevskaya, Vladimir A. Lukhtanov, Alexander V. Dantchenko, Elizaveta Yu. Gorodilova
