# Supplementary figures and images for: ﻿What is underneath? – UV analysis of wing undersides reveals high intraspecific variability in cryptic Agrodiaetus species (Lepidoptera, Lycaenidae, Polyommatus)
Source: Zookeys. 2025 Oct 20;1256:195–234. doi: 10.3897/zookeys.1256.165602 (PMC12559952; doi:10.3897/zookeys.1256.165602)

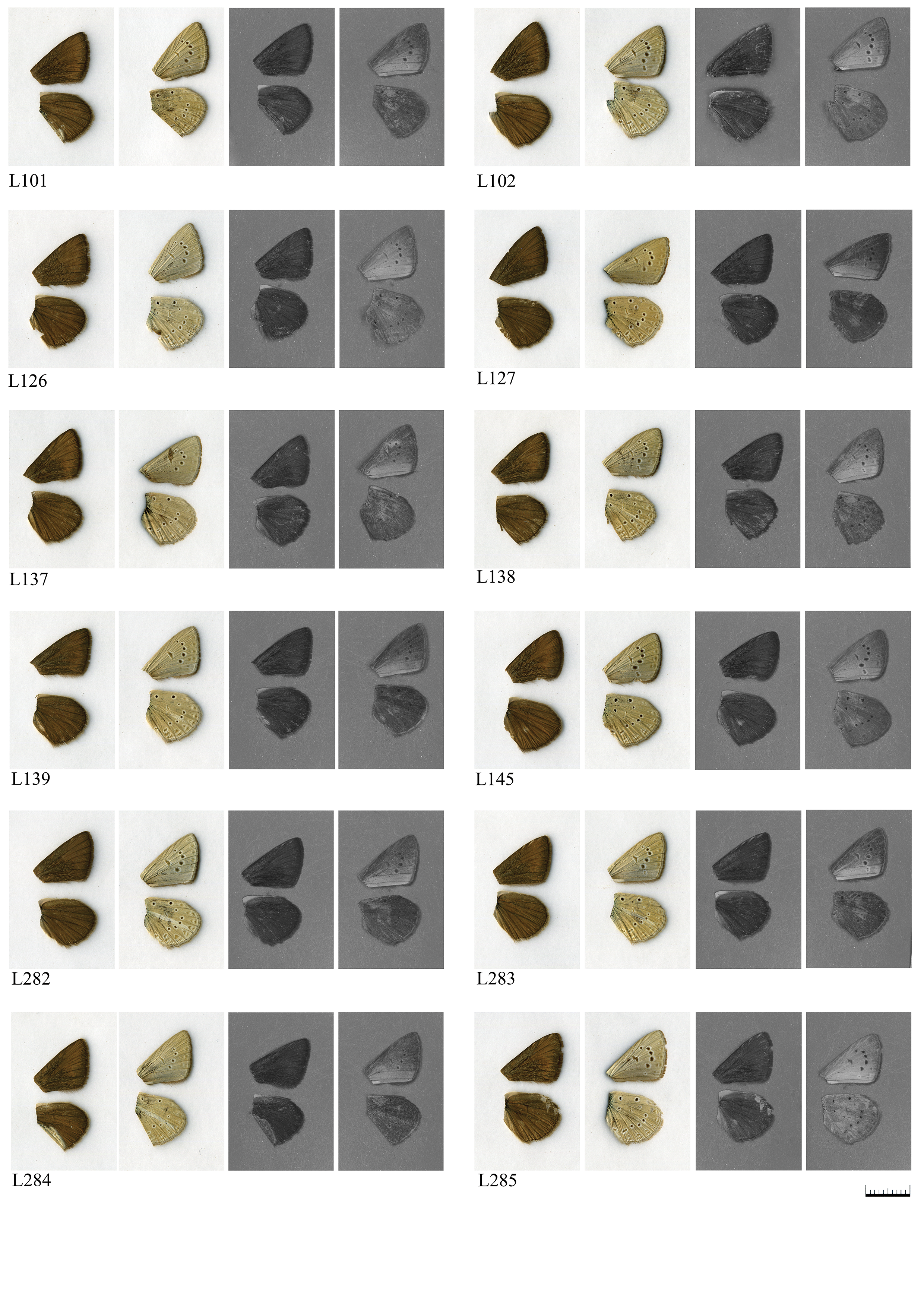

Supplement: Supplementary material 2 — Specimens of P. admetus in visible light and UV light, part 1 [file zookeys-1256-195_article-165602__-s002.tif]

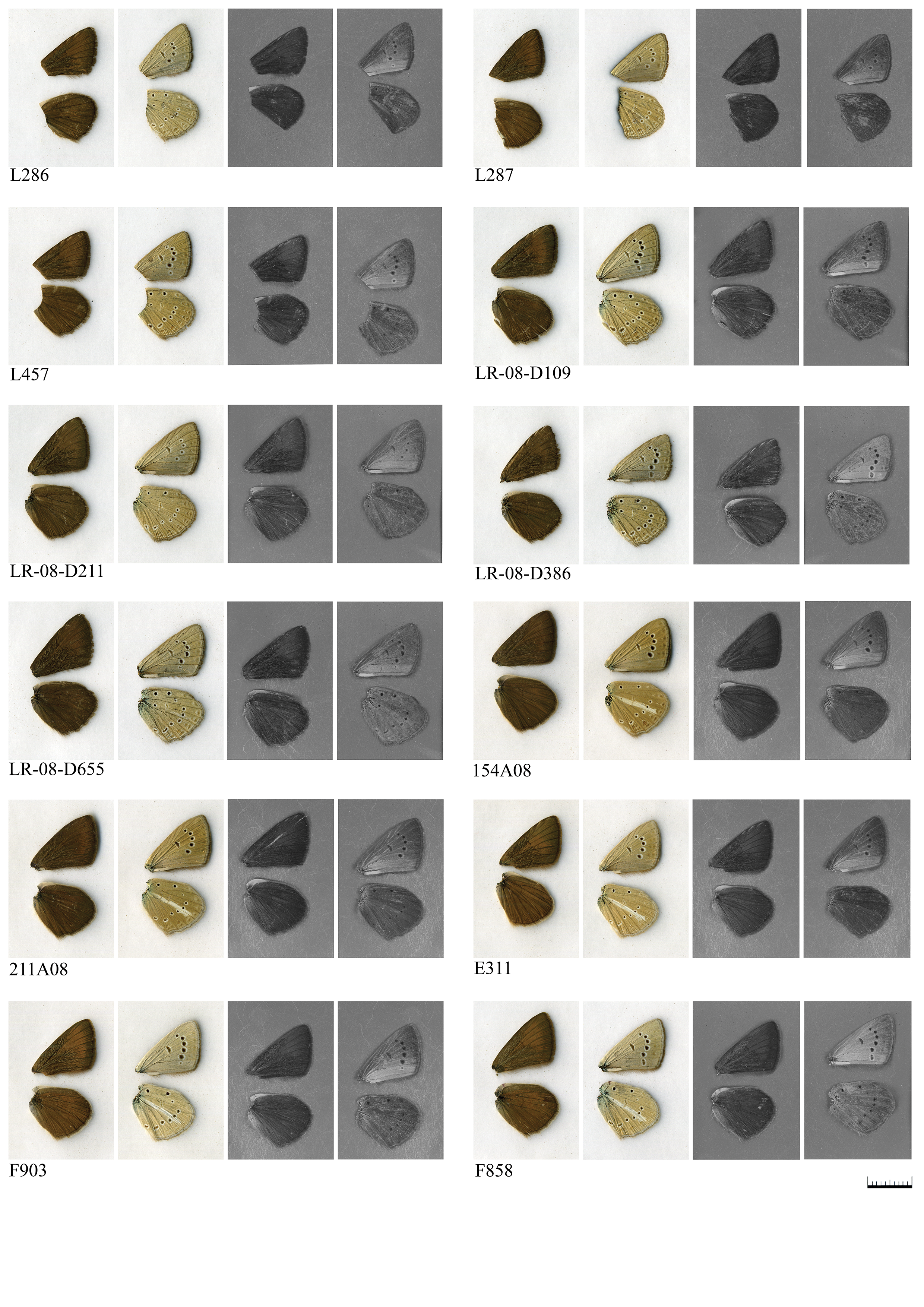

Supplement: Supplementary material 3 — Specimens of P. admetus in visible light and UV light, part 2 [file zookeys-1256-195_article-165602__-s003.tif]

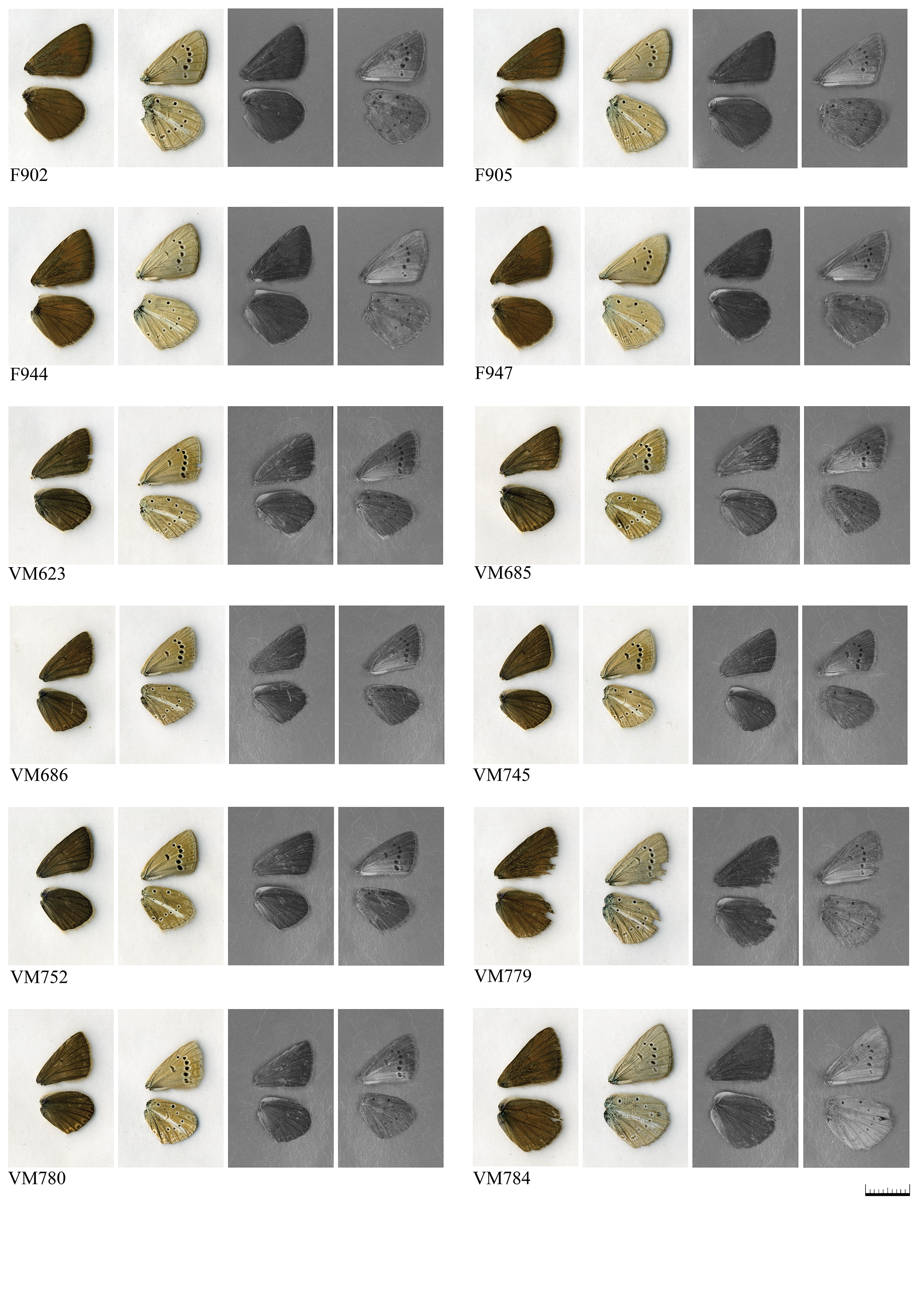

Supplement: Supplementary material 4 — Specimens of P. admetus in visible light and UV light, part 3 [file zookeys-1256-195_article-165602__-s004.tif]

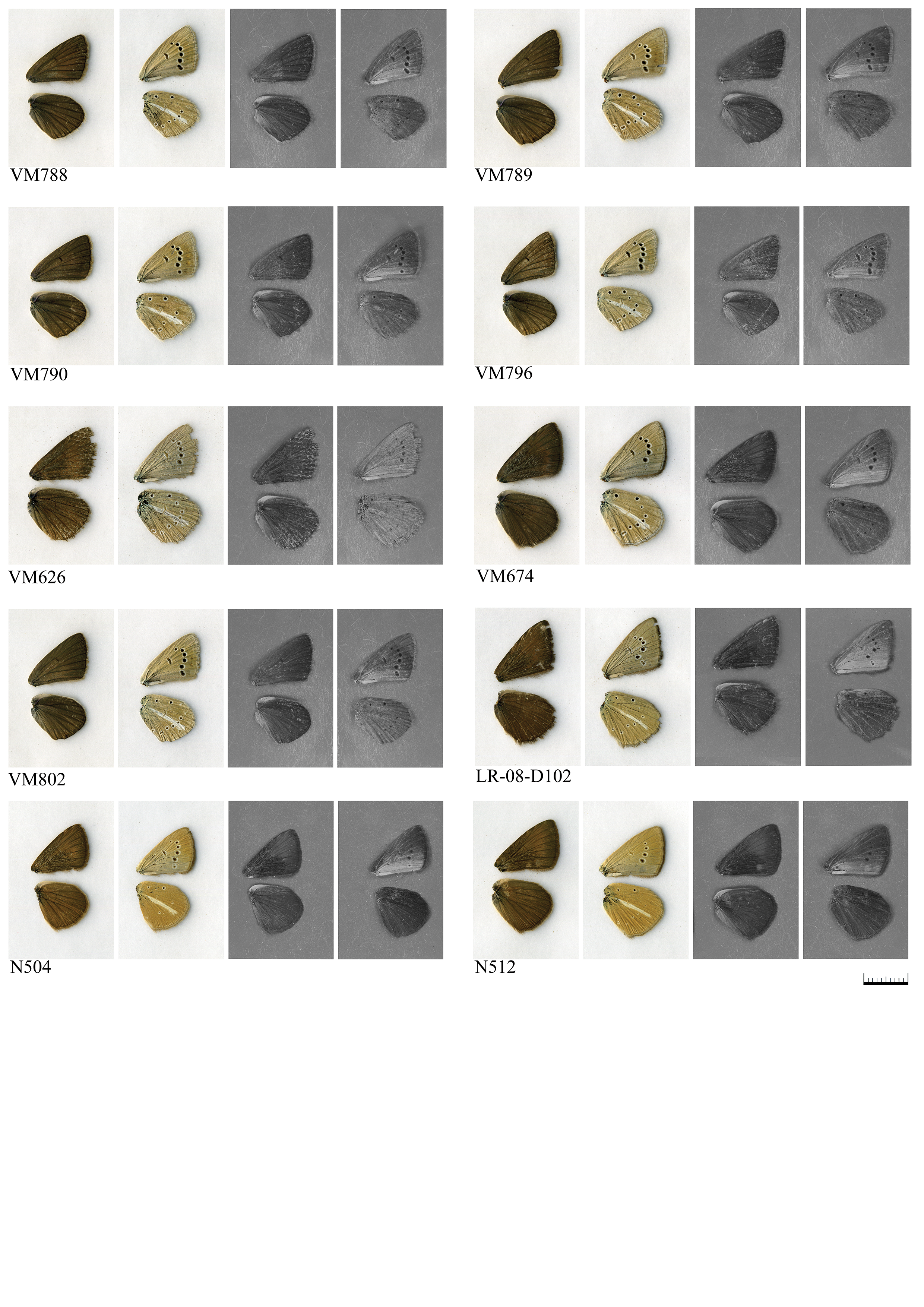

Supplement: Supplementary material 5 — Specimens of P. admetus (part 4), P. aroaniensis, P. alcestis in visible light and UV light [file zookeys-1256-195_article-165602__-s005.tif]

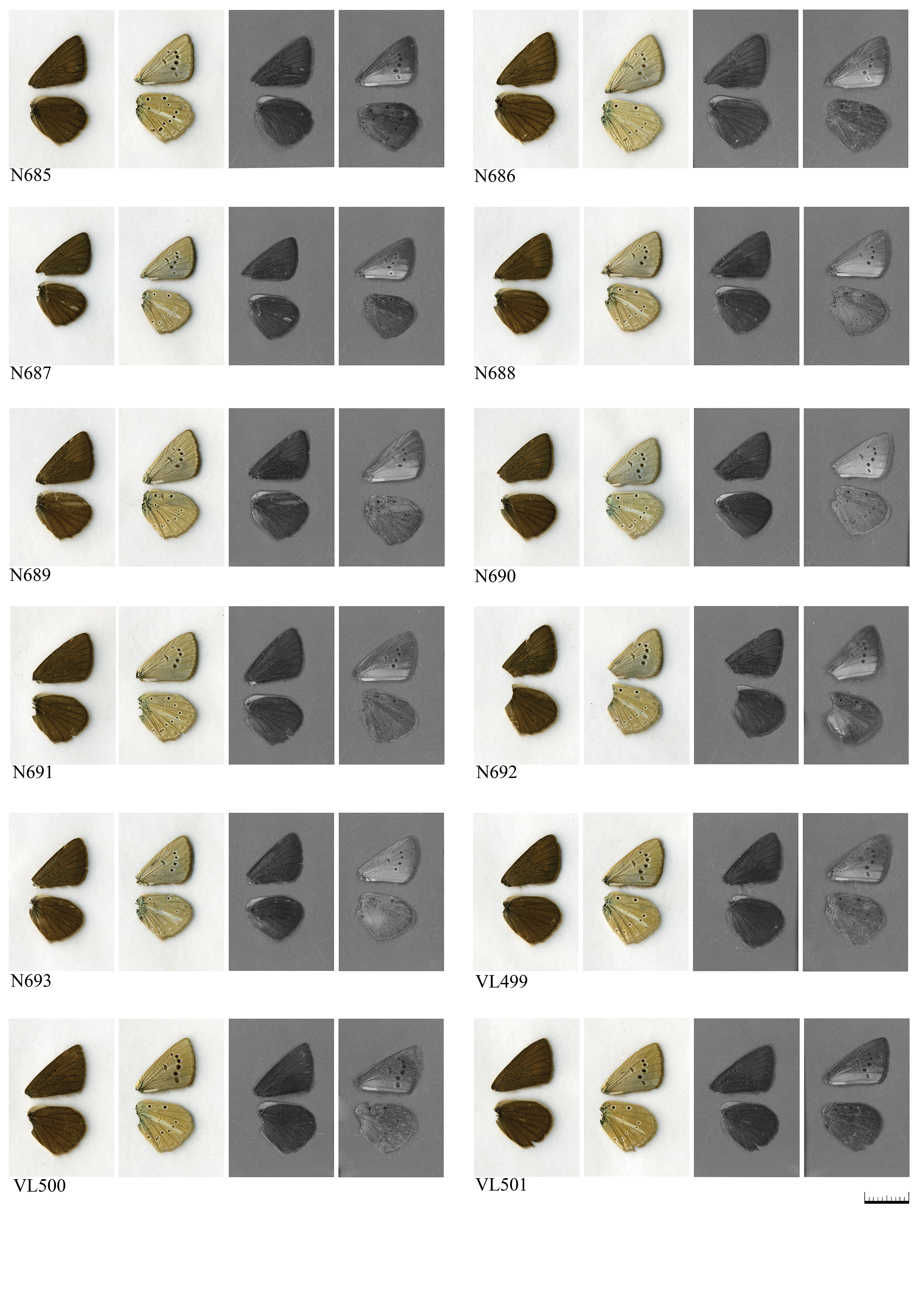

Supplement: Supplementary material 6 — Specimens of P. demavendi in visible light and UV light, part 1 [file zookeys-1256-195_article-165602__-s006.tif]

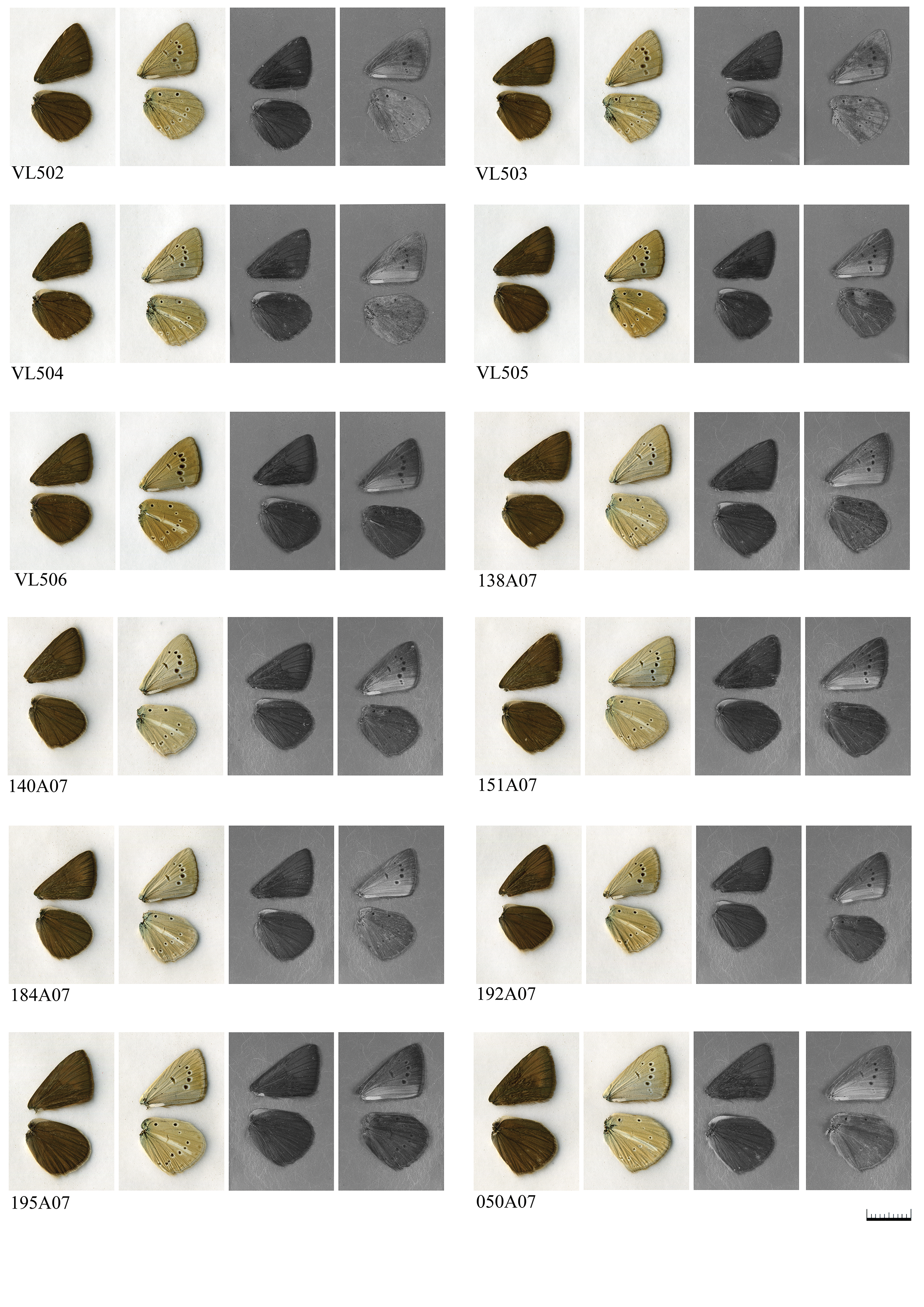

Supplement: Supplementary material 7 — Specimens of P. demavendi in visible light and UV light, part 2 [file zookeys-1256-195_article-165602__-s007.tif]

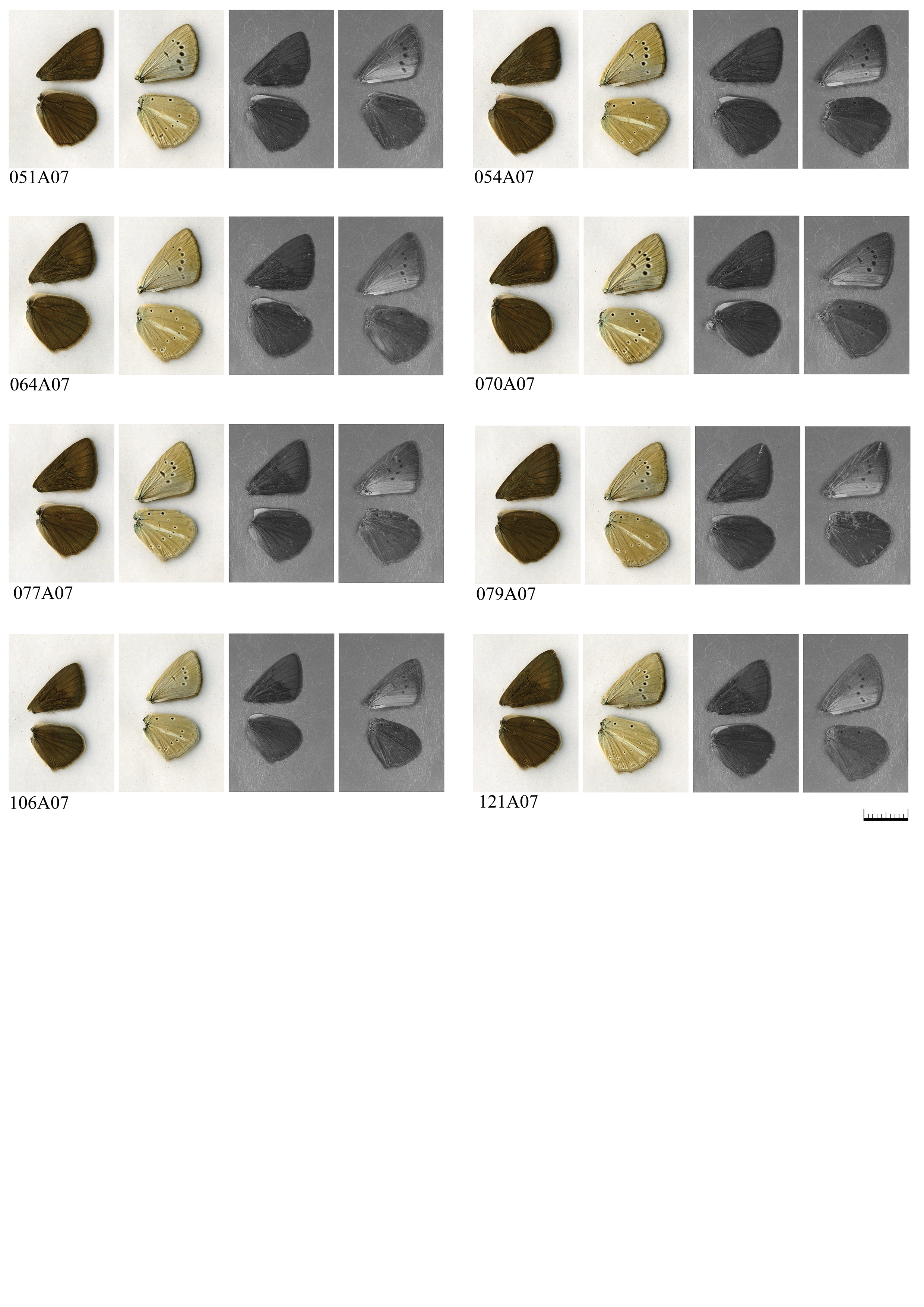

Supplement: Supplementary material 8 — Specimens of P. demavendi in visible light and UV light, part 3 [file zookeys-1256-195_article-165602__-s008.tif]

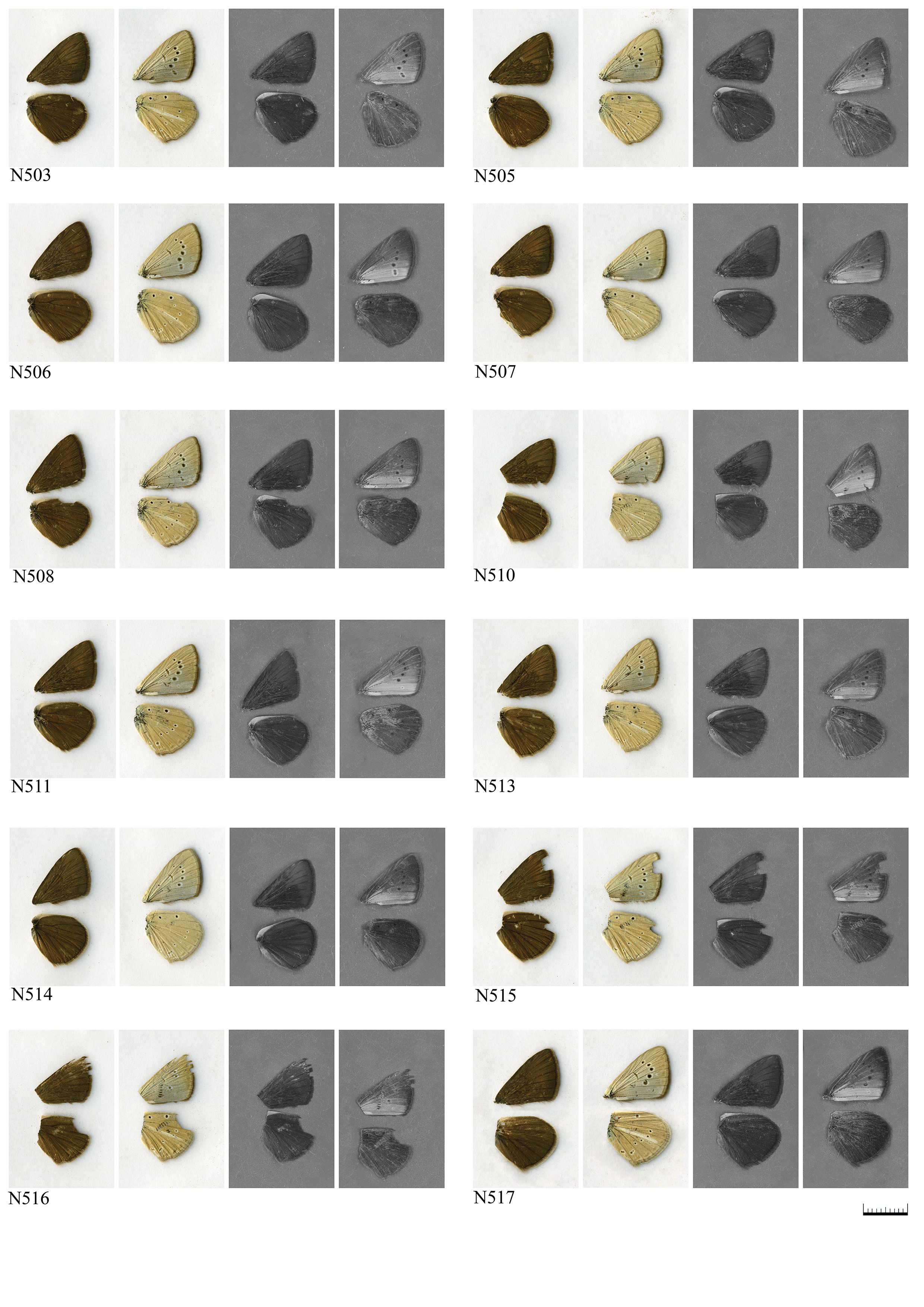

Supplement: Supplementary material 9 — Specimens of P. demavendi lorestanus in visible light and UV light, part 1 [file zookeys-1256-195_article-165602__-s009.tif]

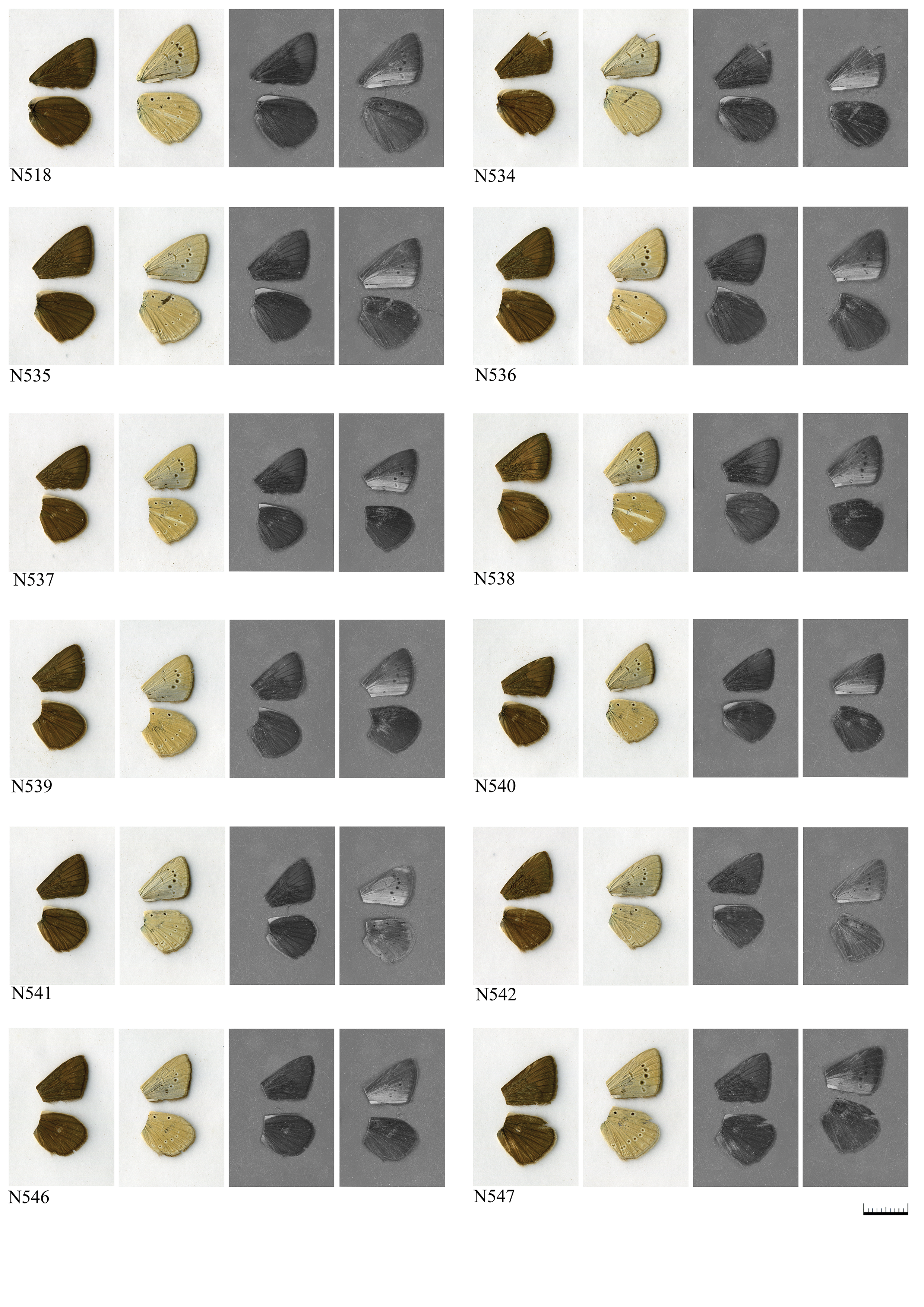

Supplement: Supplementary material 10 — Specimens of P. demavendi lorestanus in visible light and UV light, part 2 [file zookeys-1256-195_article-165602__-s010.tif]

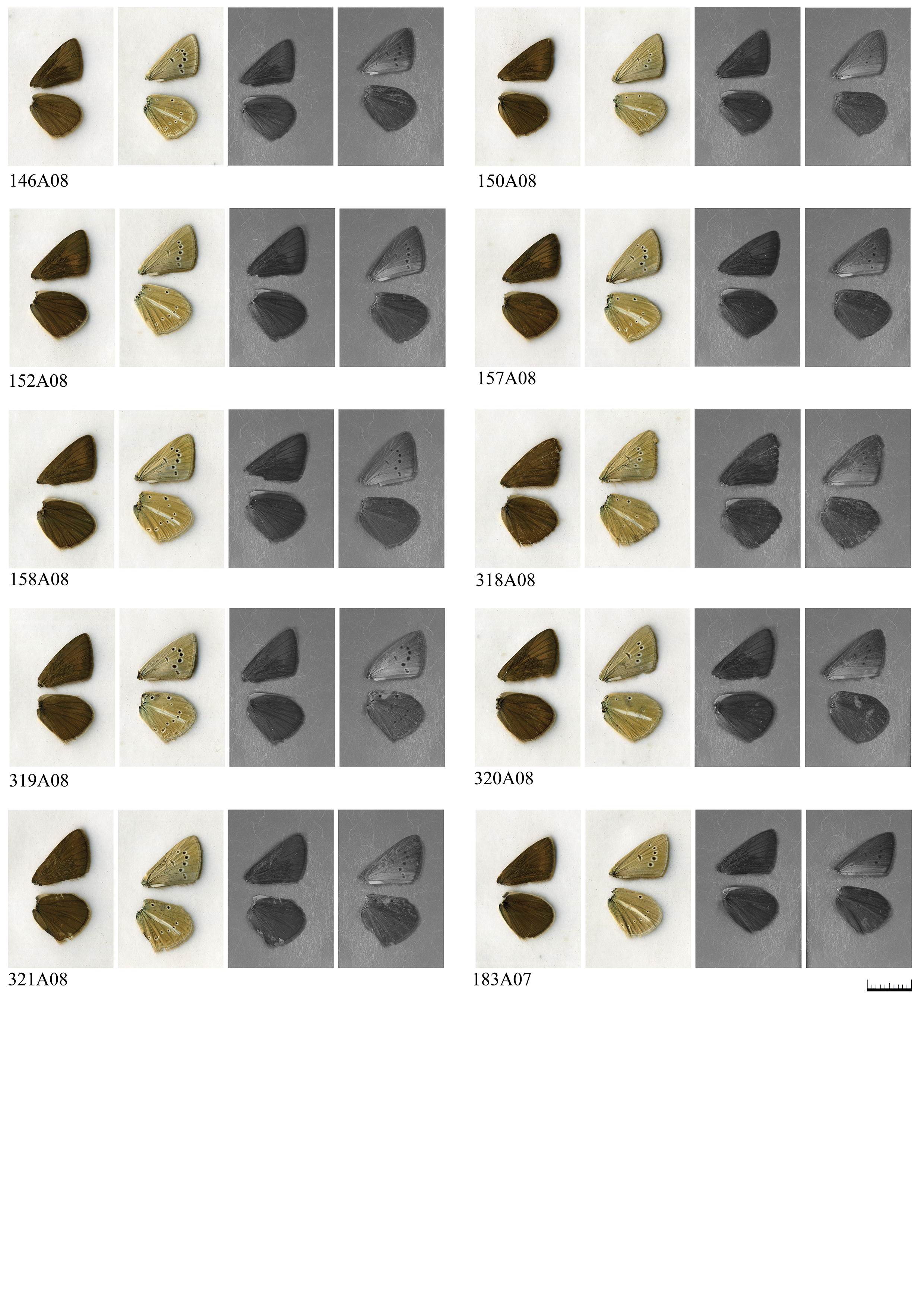

Supplement: Supplementary material 11 — Specimens of P. emmeli in visible light and UV light [file zookeys-1256-195_article-165602__-s011.tif]

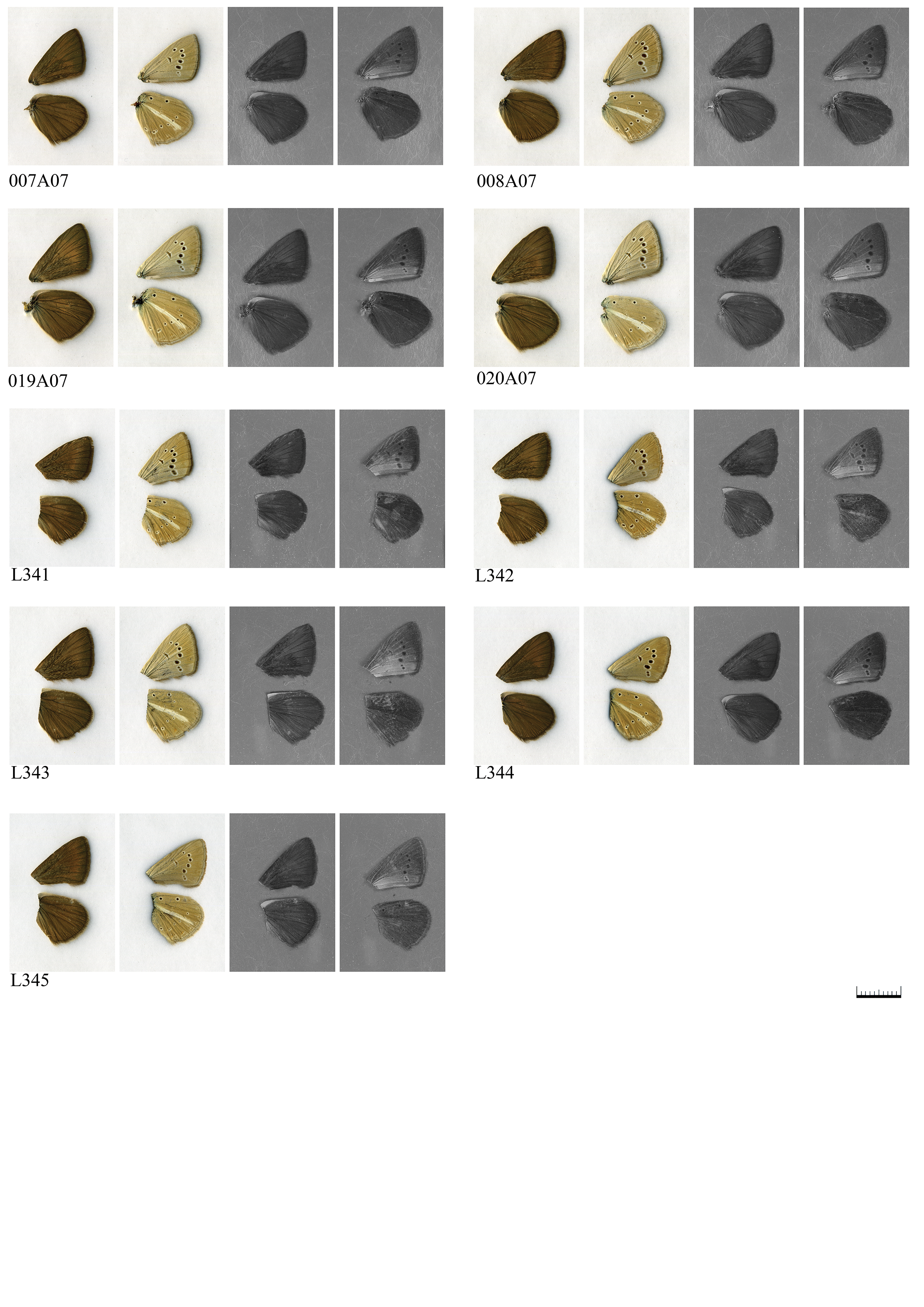

Supplement: Supplementary material 12 — Specimens of P. eriwanensis and P. dantchenkoi in visible light and UV light [file zookeys-1256-195_article-165602__-s012.tif]

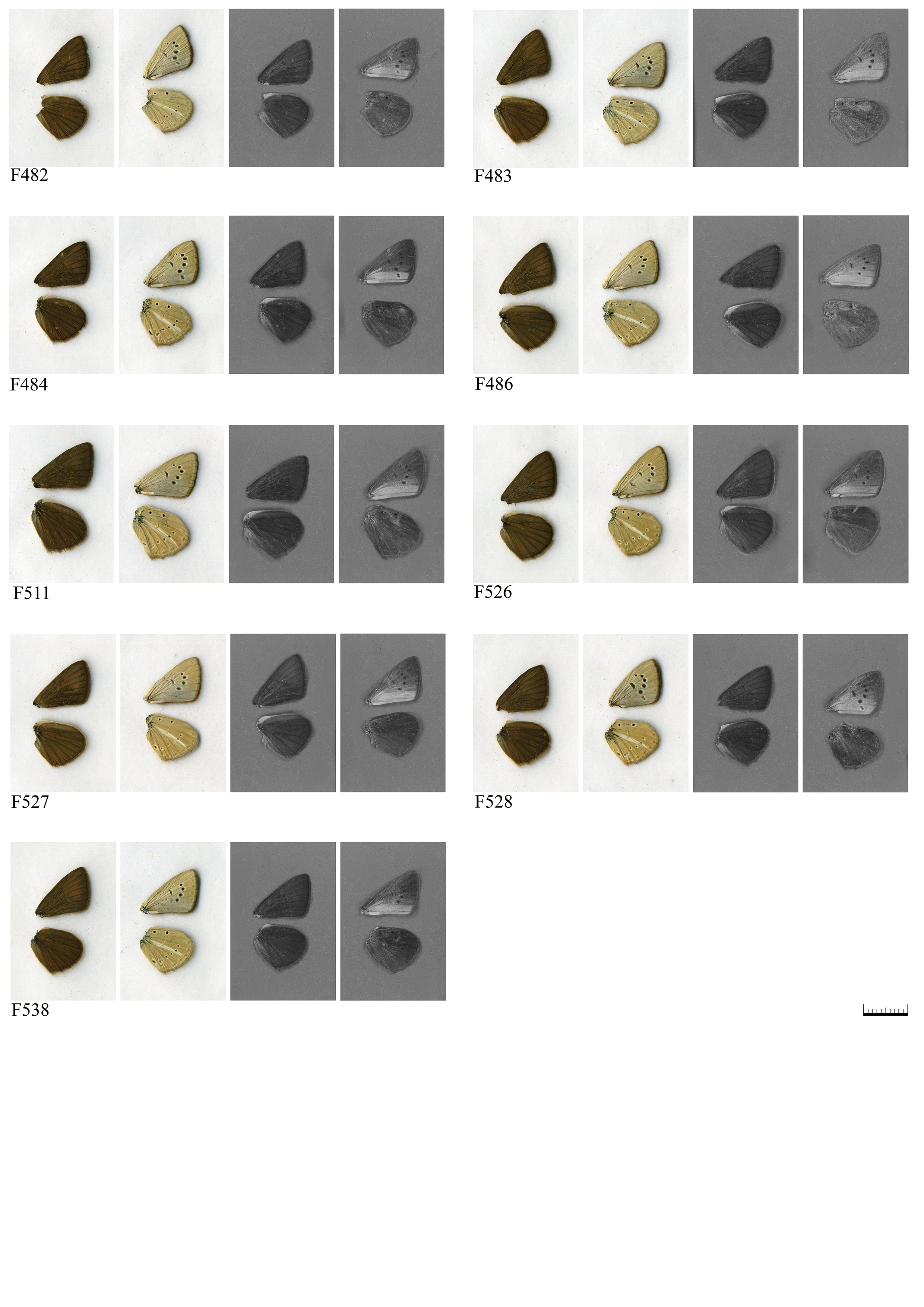

Supplement: Supplementary material 13 — Specimens of P. khorasanensis in visible light and UV light [file zookeys-1256-195_article-165602__-s013.tif]

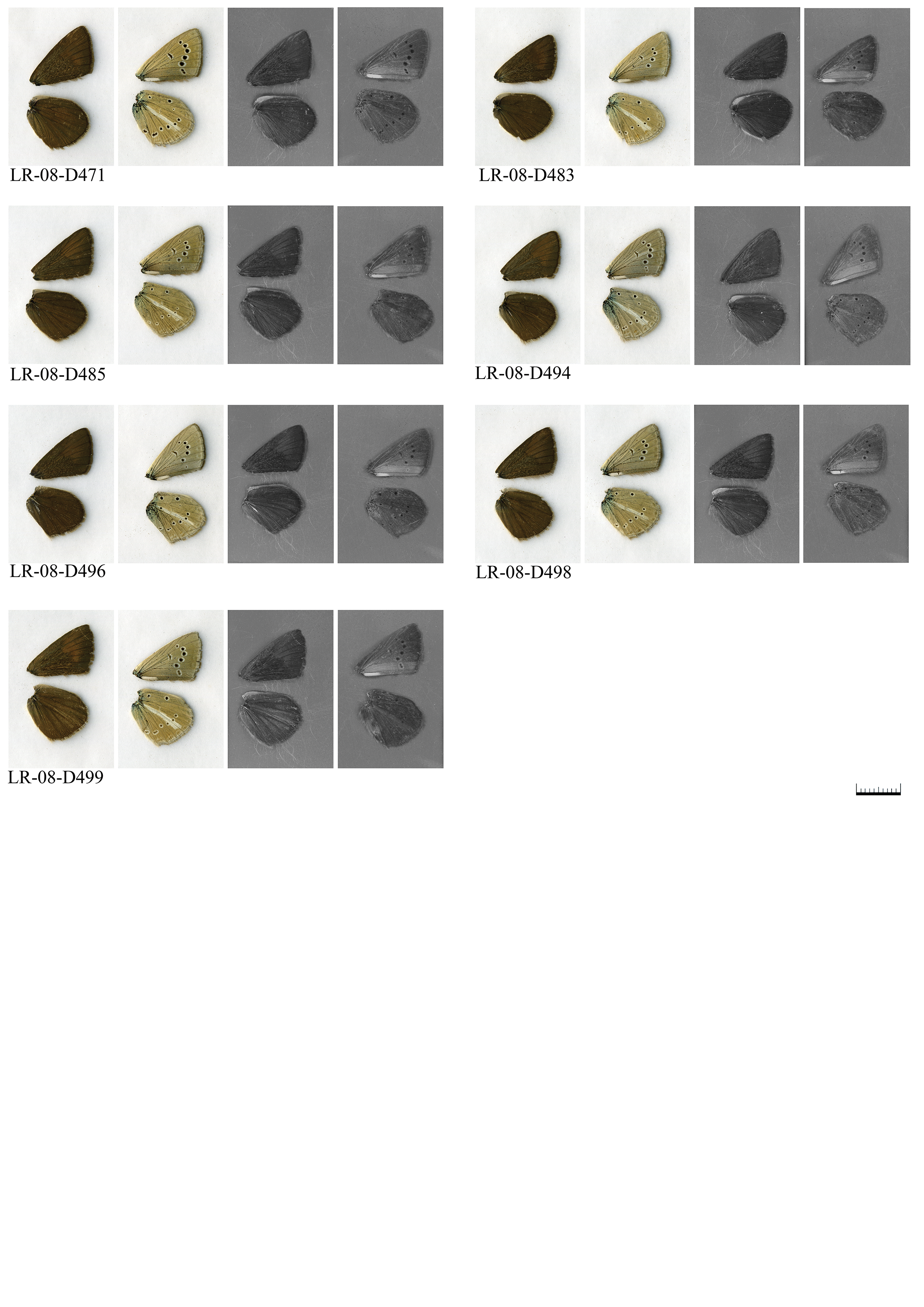

Supplement: Supplementary material 14 — Specimens of P. nephohiptamenos in visible light and UV light [file zookeys-1256-195_article-165602__-s014.tif]

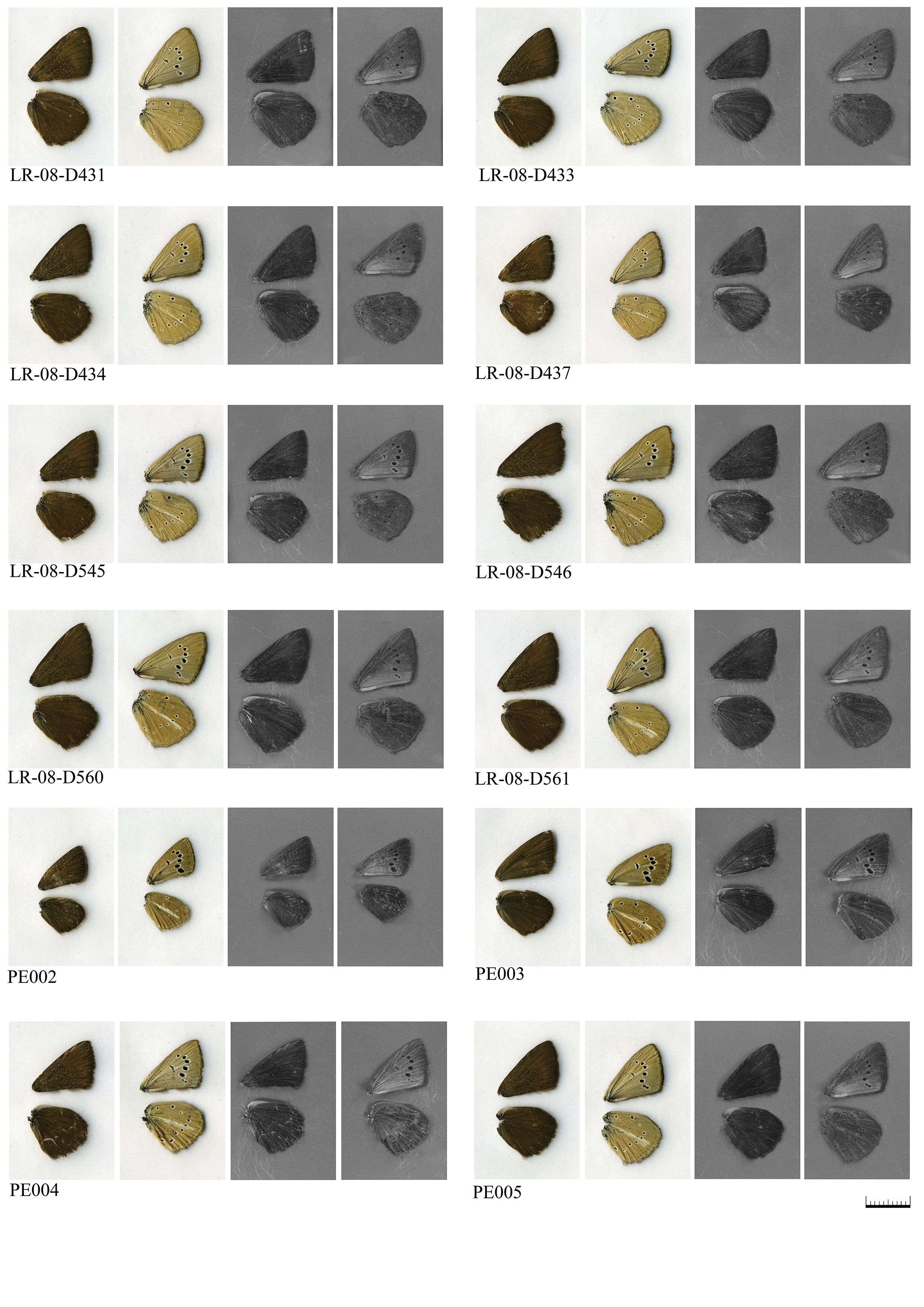

Supplement: Supplementary material 15 — Specimens of P. orphicus in visible light and UV light, part 1 [file zookeys-1256-195_article-165602__-s015.tif]

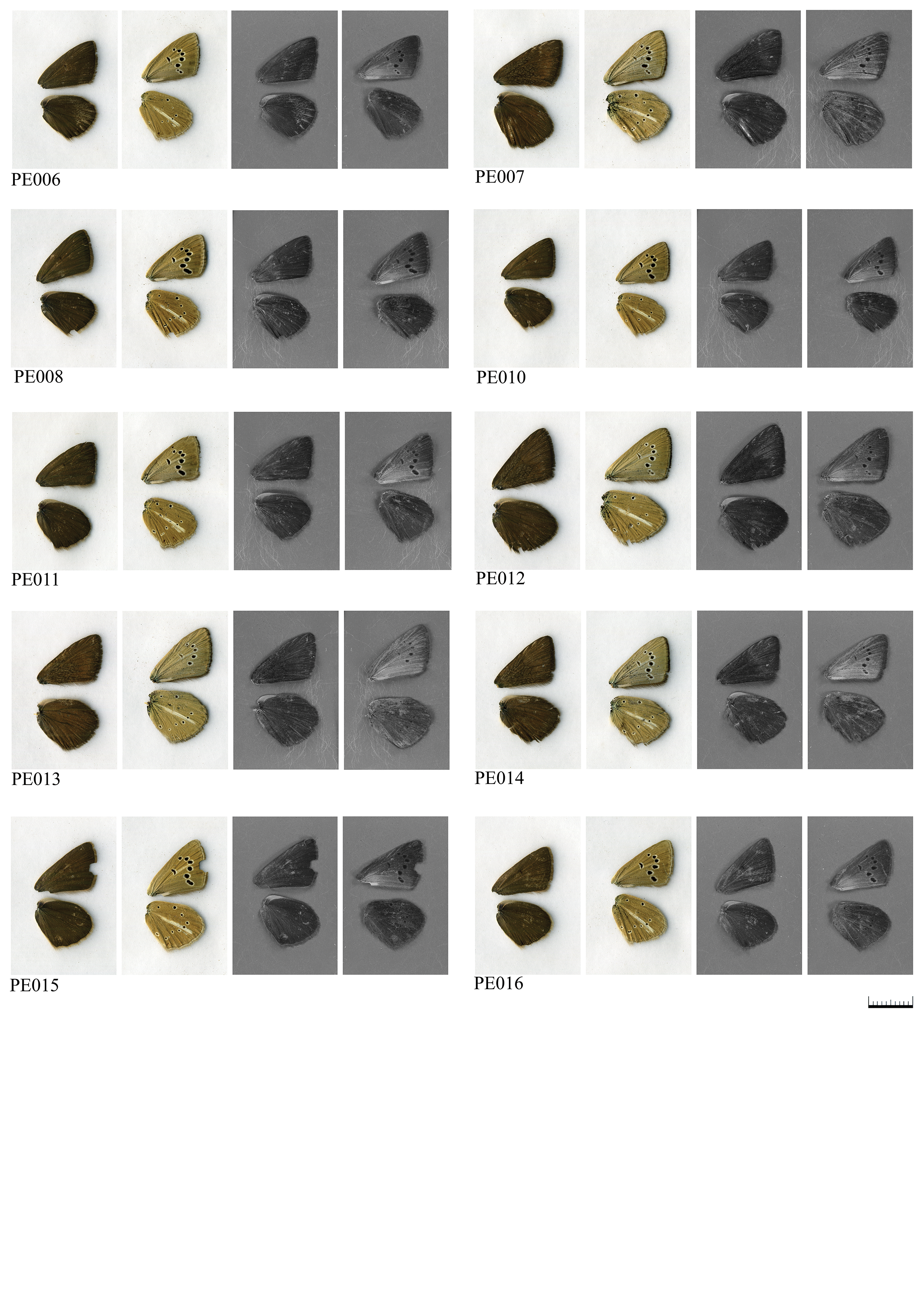

Supplement: Supplementary material 16 — Specimens of P. orphicus in visible light and UV light, part 2 [file zookeys-1256-195_article-165602__-s016.tif]

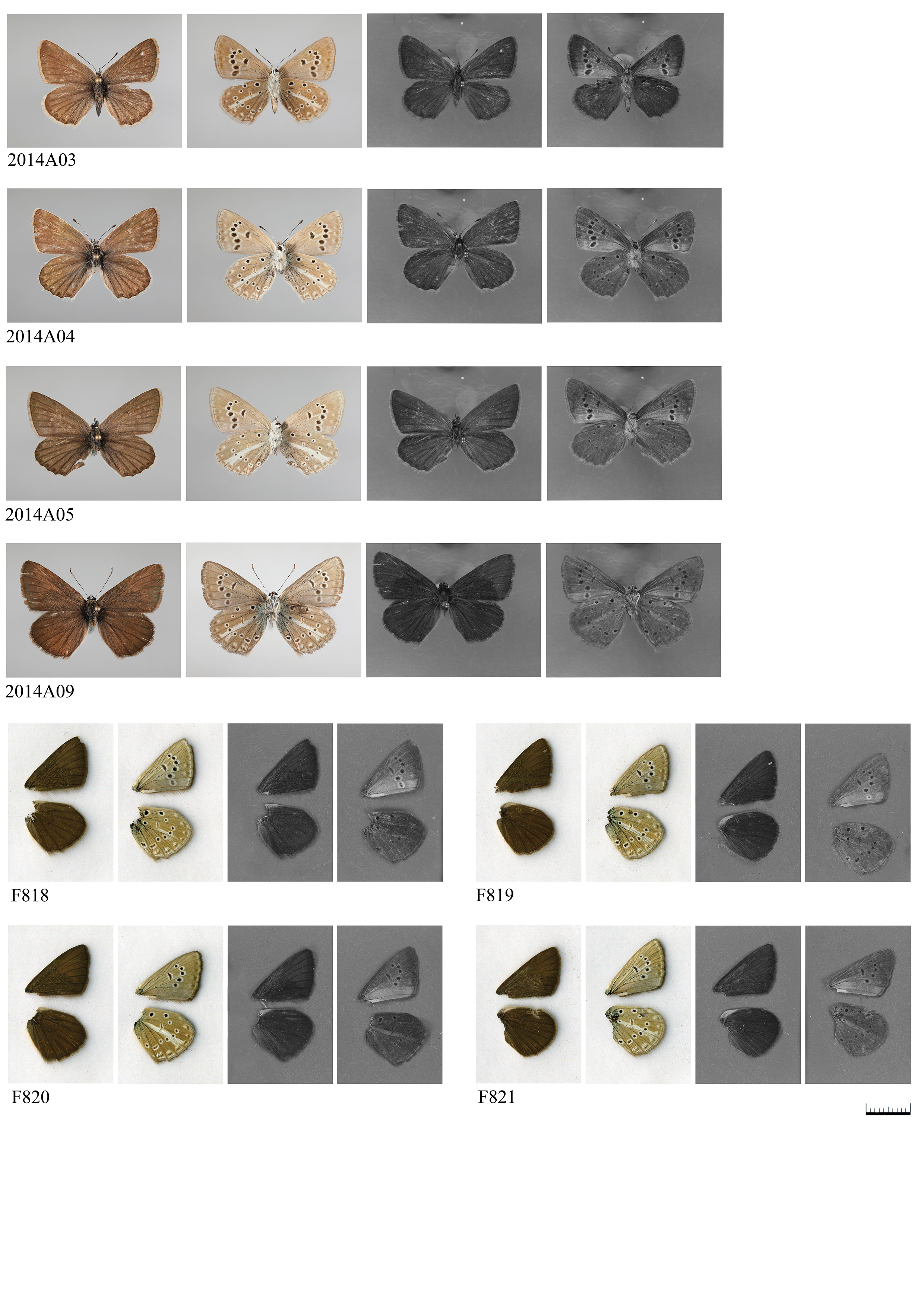

Supplement: Supplementary material 17 — Specimens of P. pseudorjabovi in visible light and UV light, part 1 [file zookeys-1256-195_article-165602__-s017.tif]

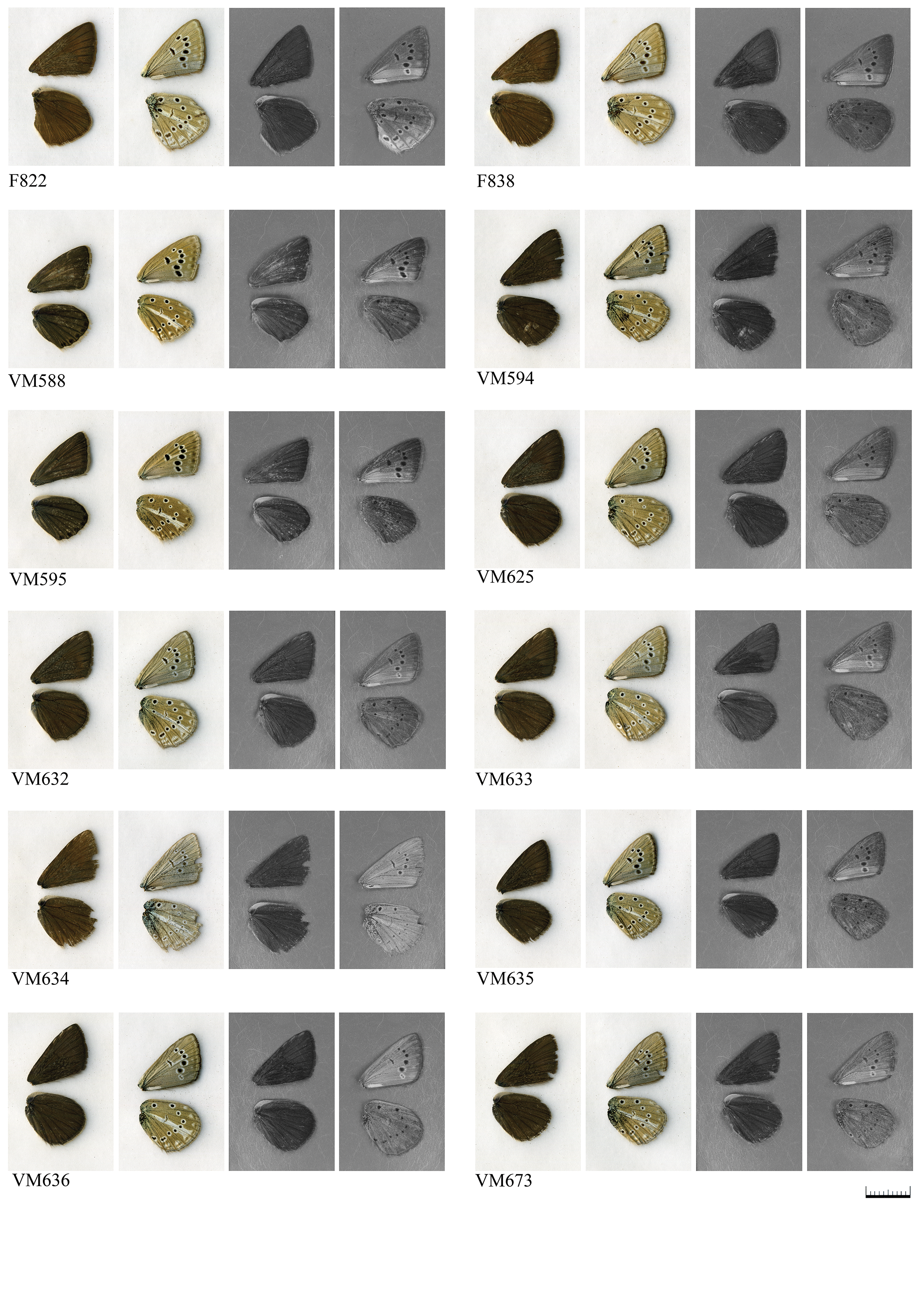

Supplement: Supplementary material 18 — Specimens of P. pseudorjabovi in visible light and UV light, part 2 [file zookeys-1256-195_article-165602__-s018.tif]

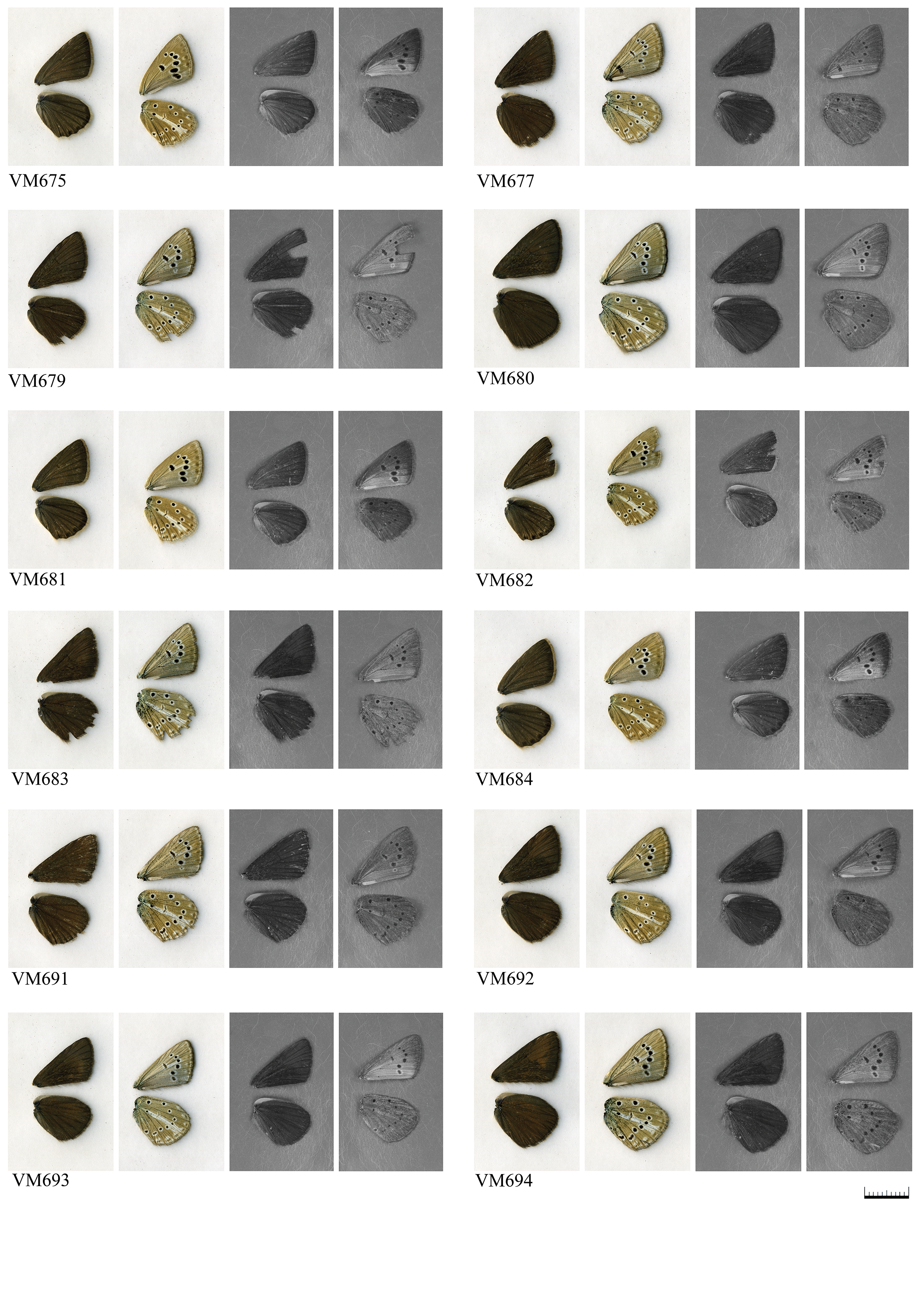

Supplement: Supplementary material 19 — Specimens of P. pseudorjabovi in visible light and UV light, part 3 [file zookeys-1256-195_article-165602__-s019.tif]

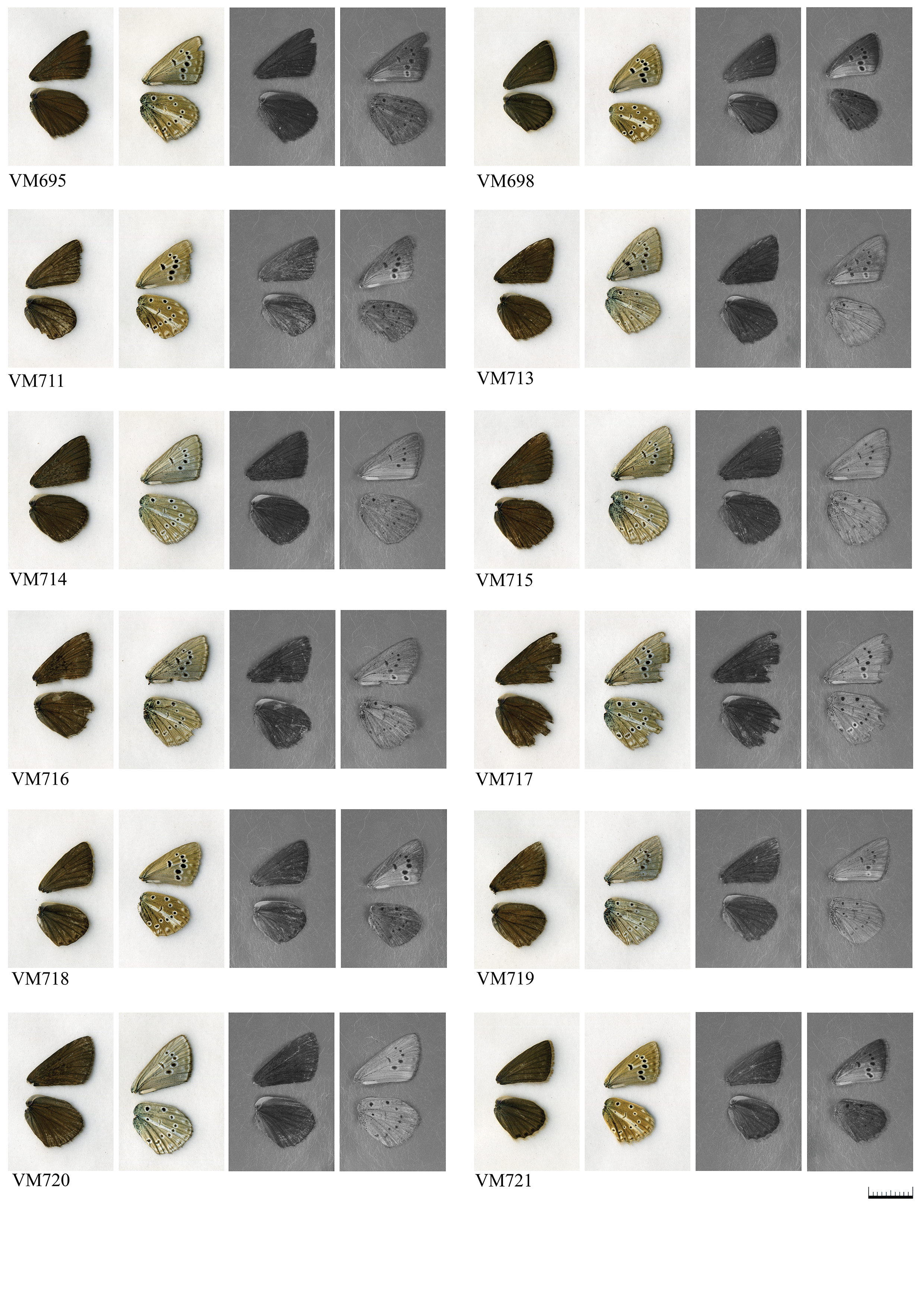

Supplement: Supplementary material 20 — Specimens of P. pseudorjabovi in visible light and UV light, part 4 [file zookeys-1256-195_article-165602__-s020.tif]

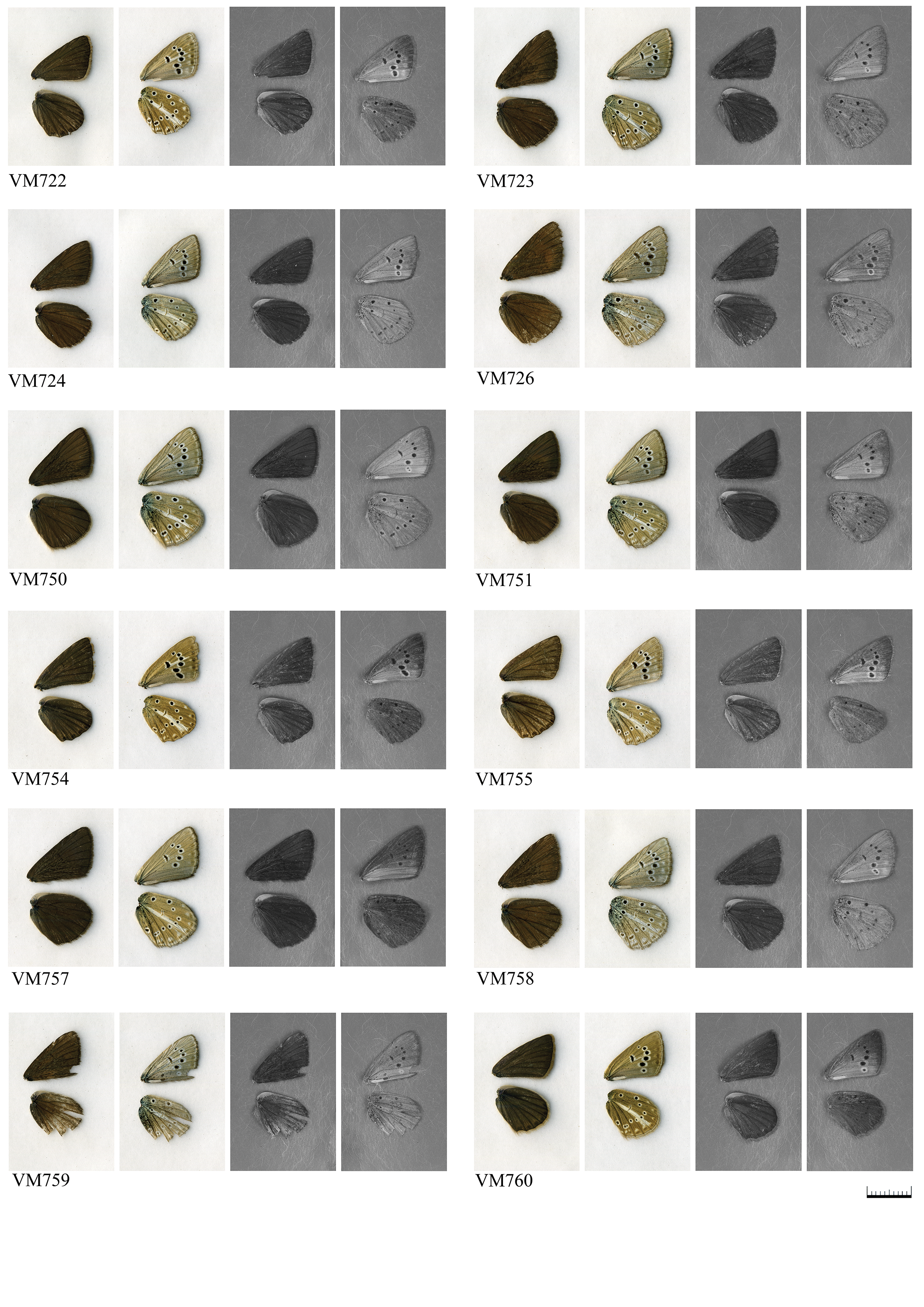

Supplement: Supplementary material 21 — Specimens of P. pseudorjabovi in visible light and UV light, part 5 [file zookeys-1256-195_article-165602__-s021.tif]

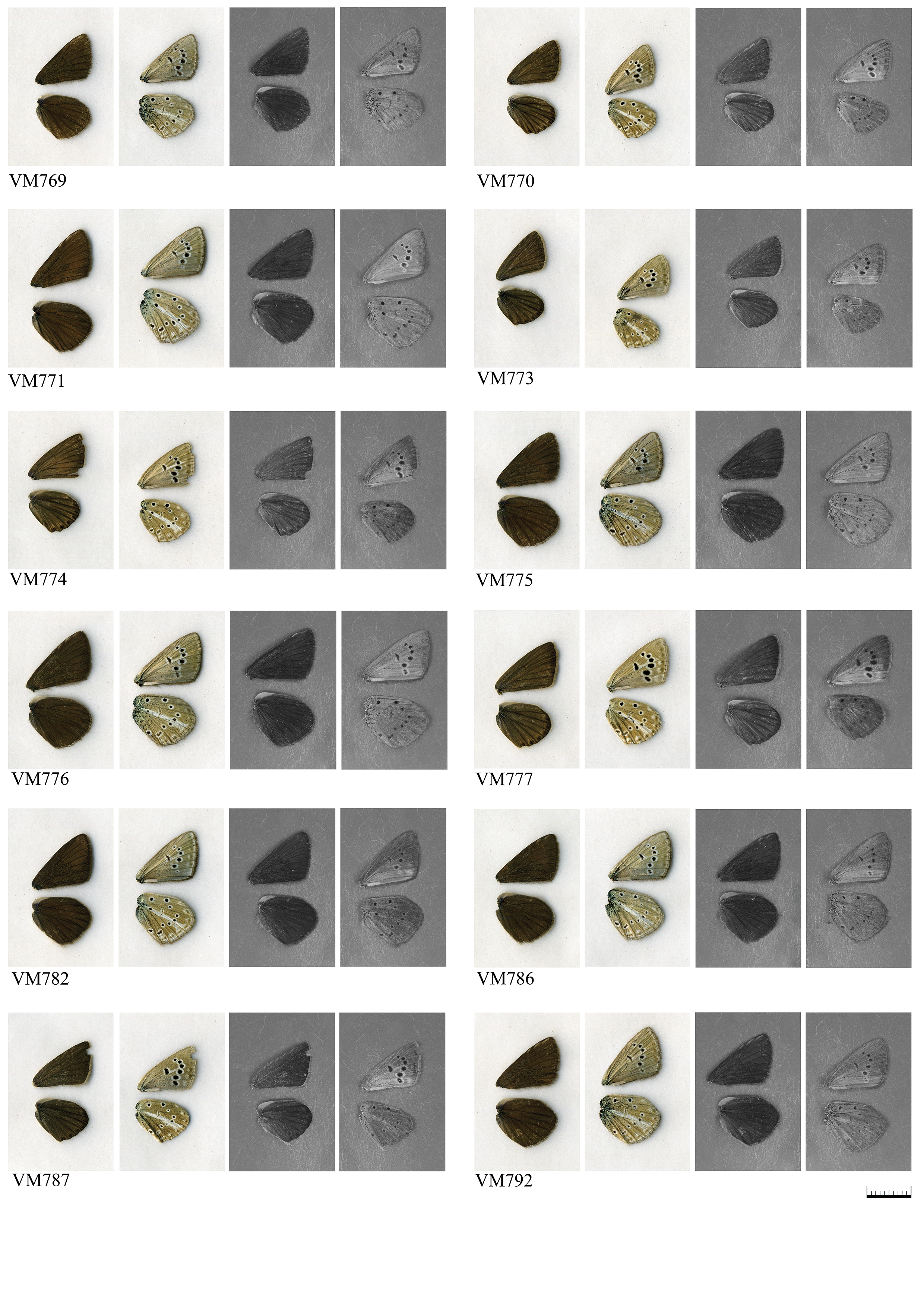

Supplement: Supplementary material 22 — Specimens of P. pseudorjabovi in visible light and UV light, part 6 [file zookeys-1256-195_article-165602__-s022.tif]

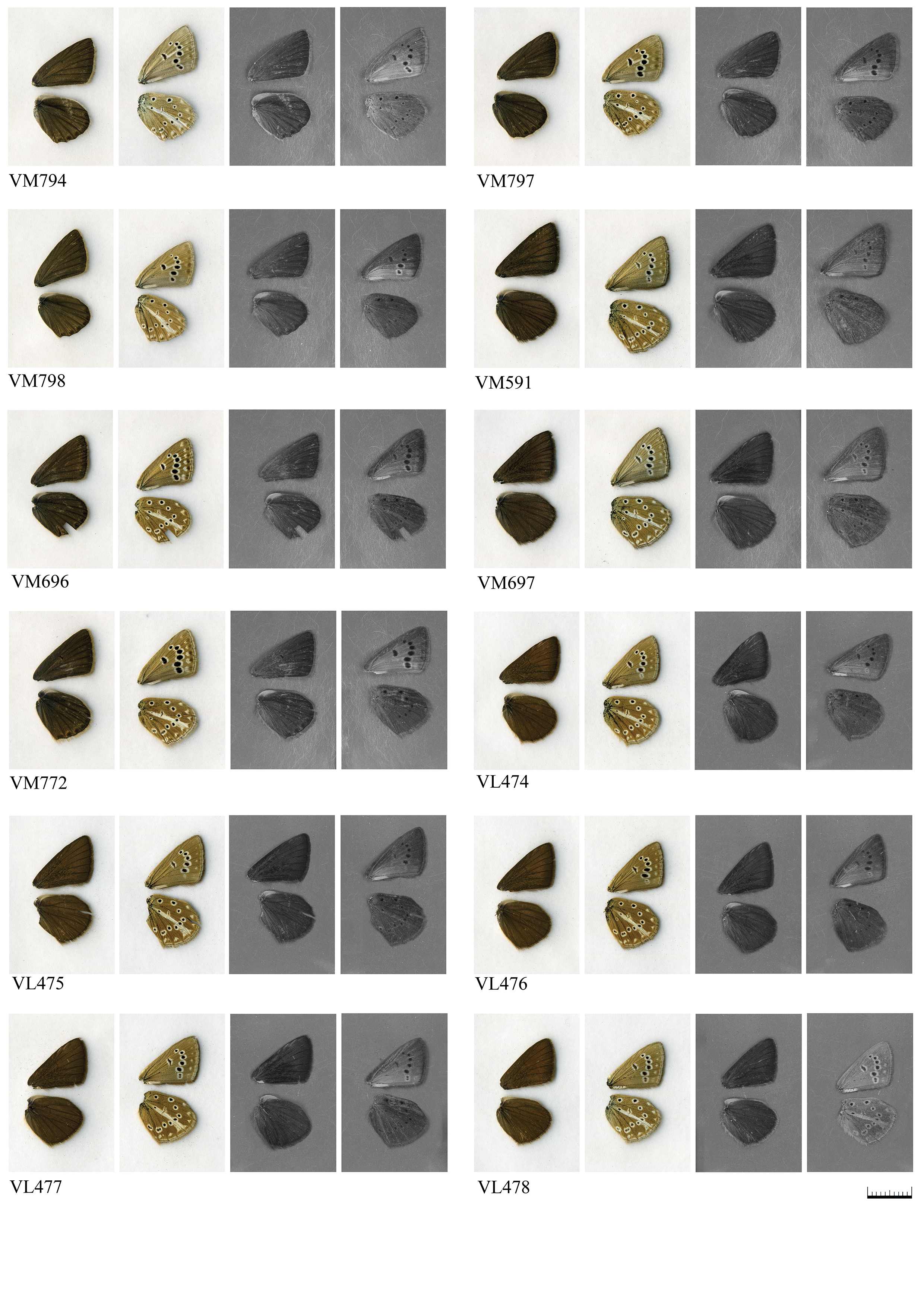

Supplement: Supplementary material 23 — Specimens of P. pseudorjabovi (part 7) and P. rjabovianus (part 1) in visible light and UV light [file zookeys-1256-195_article-165602__-s023.tif]

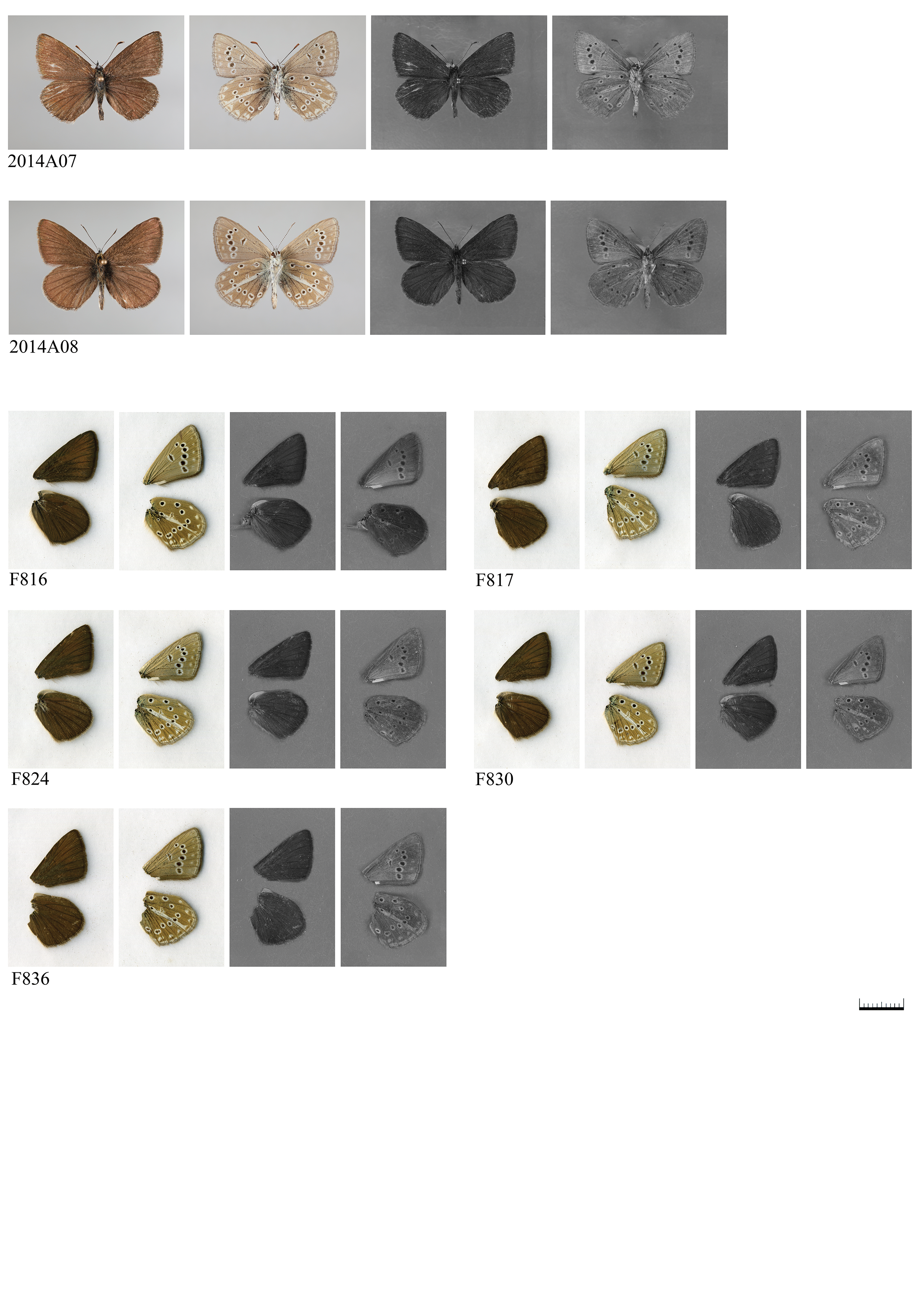

Supplement: Supplementary material 24 — Specimens of P. rjabovianus in visible light and UV light, part 2 [file zookeys-1256-195_article-165602__-s024.tif]

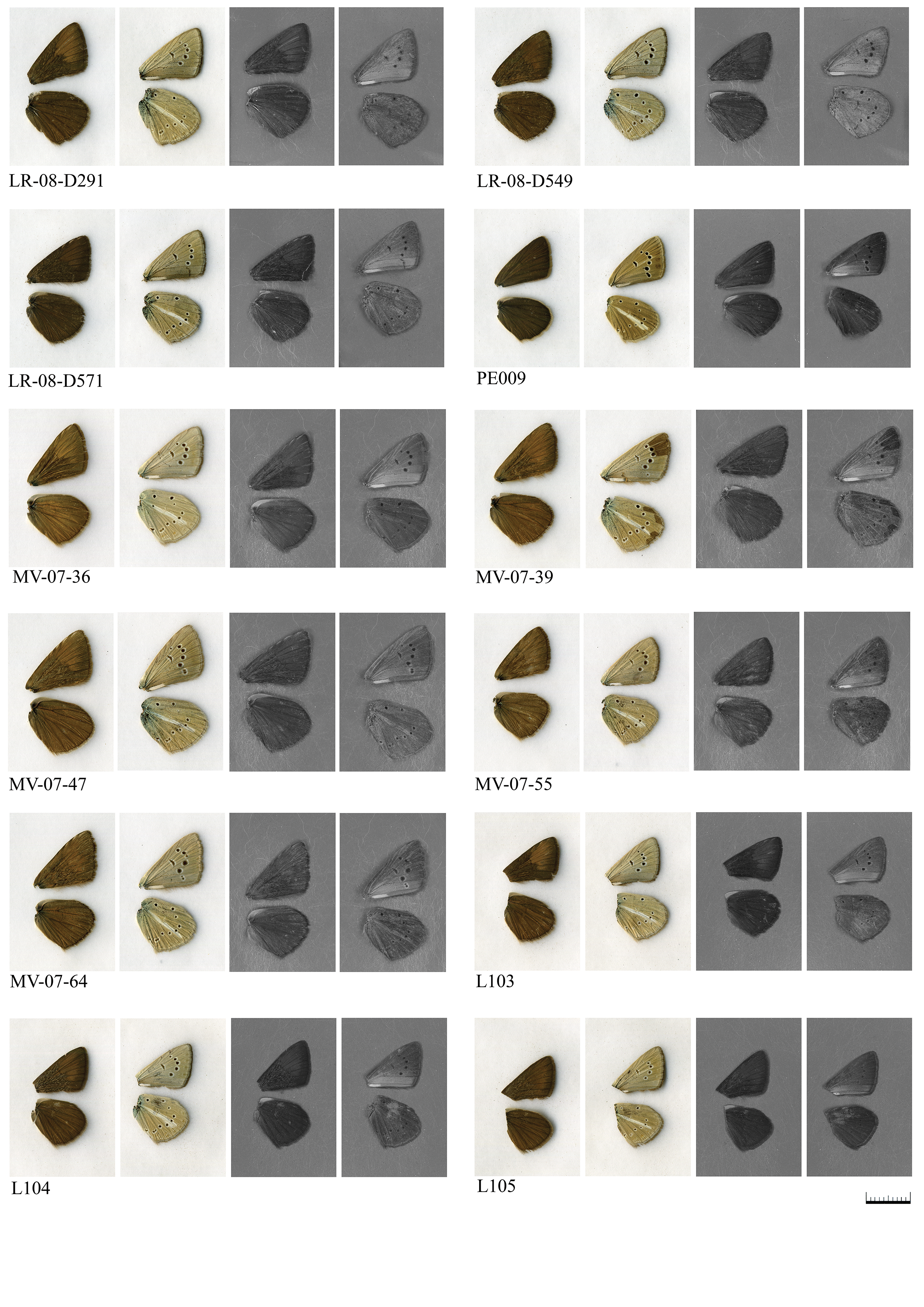

Supplement: Supplementary material 25 — Specimens of P. ripartii, in visible light and UV light, part 1 [file zookeys-1256-195_article-165602__-s025.tif]

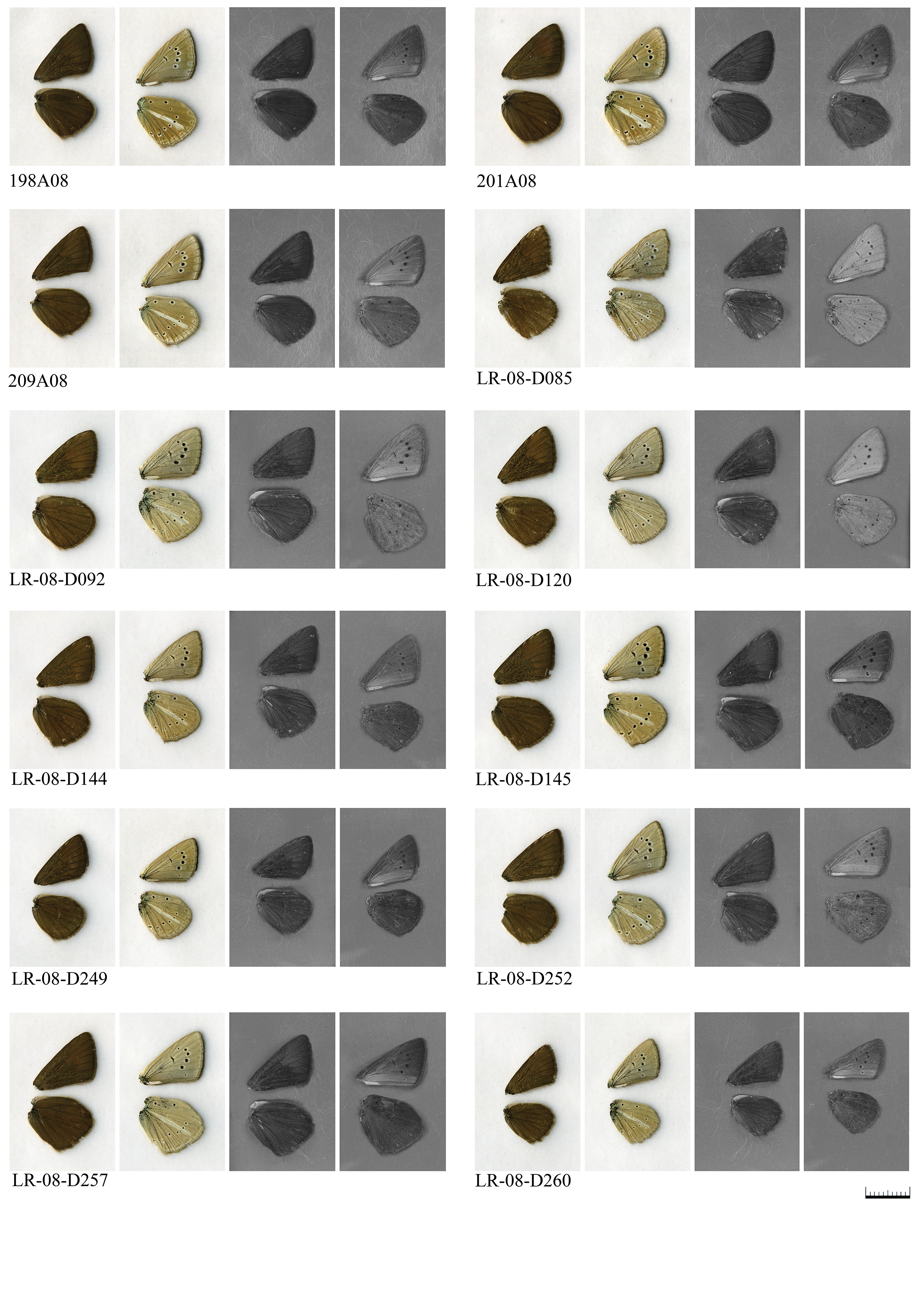

Supplement: Supplementary material 26 — Specimens of P. ripartii, in visible light and UV light, part 2 [file zookeys-1256-195_article-165602__-s026.tif]

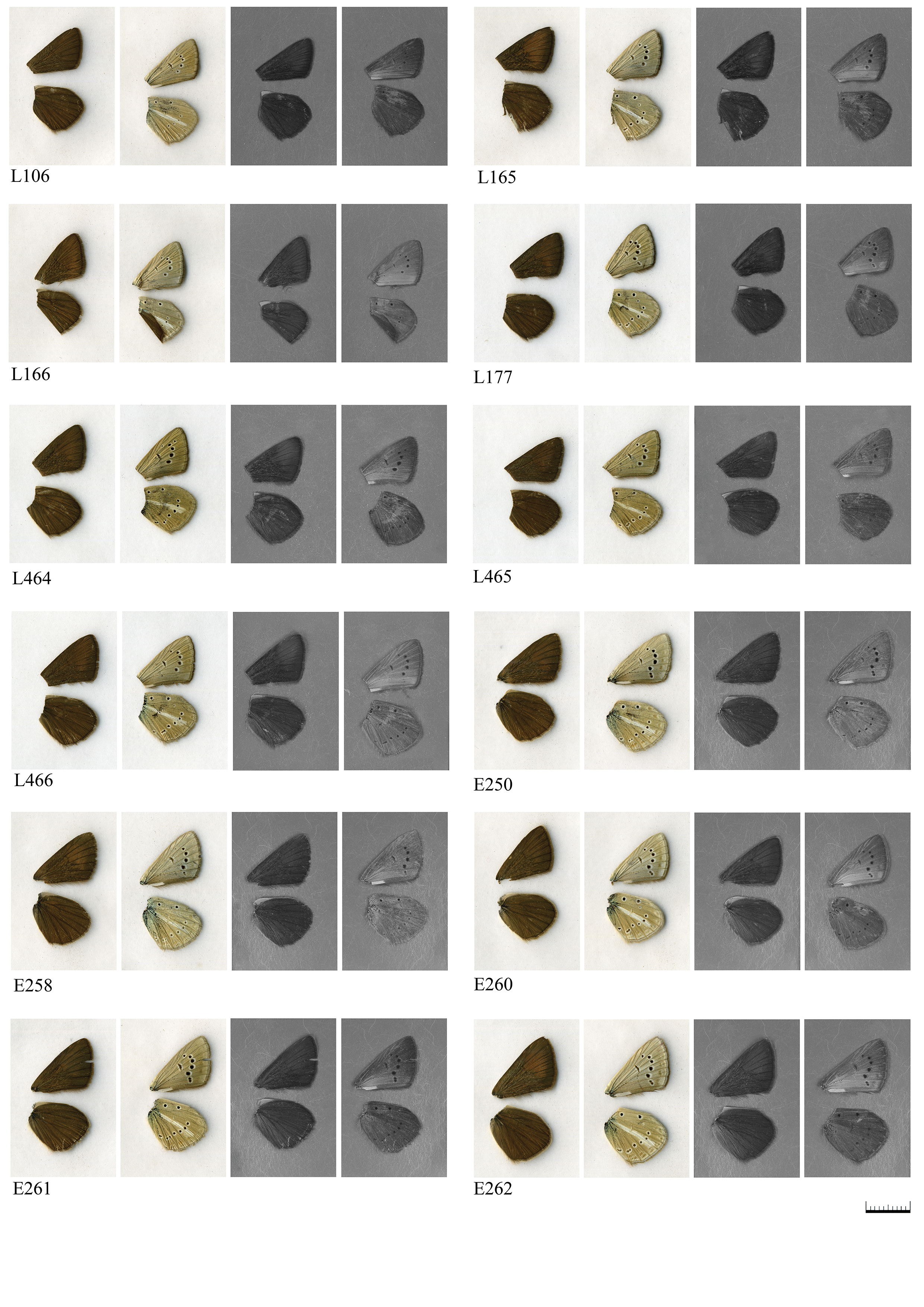

Supplement: Supplementary material 27 — Specimens of P. ripartii (part 3) and P. keleybaricus in visible light and UV light [file zookeys-1256-195_article-165602__-s027.tif]

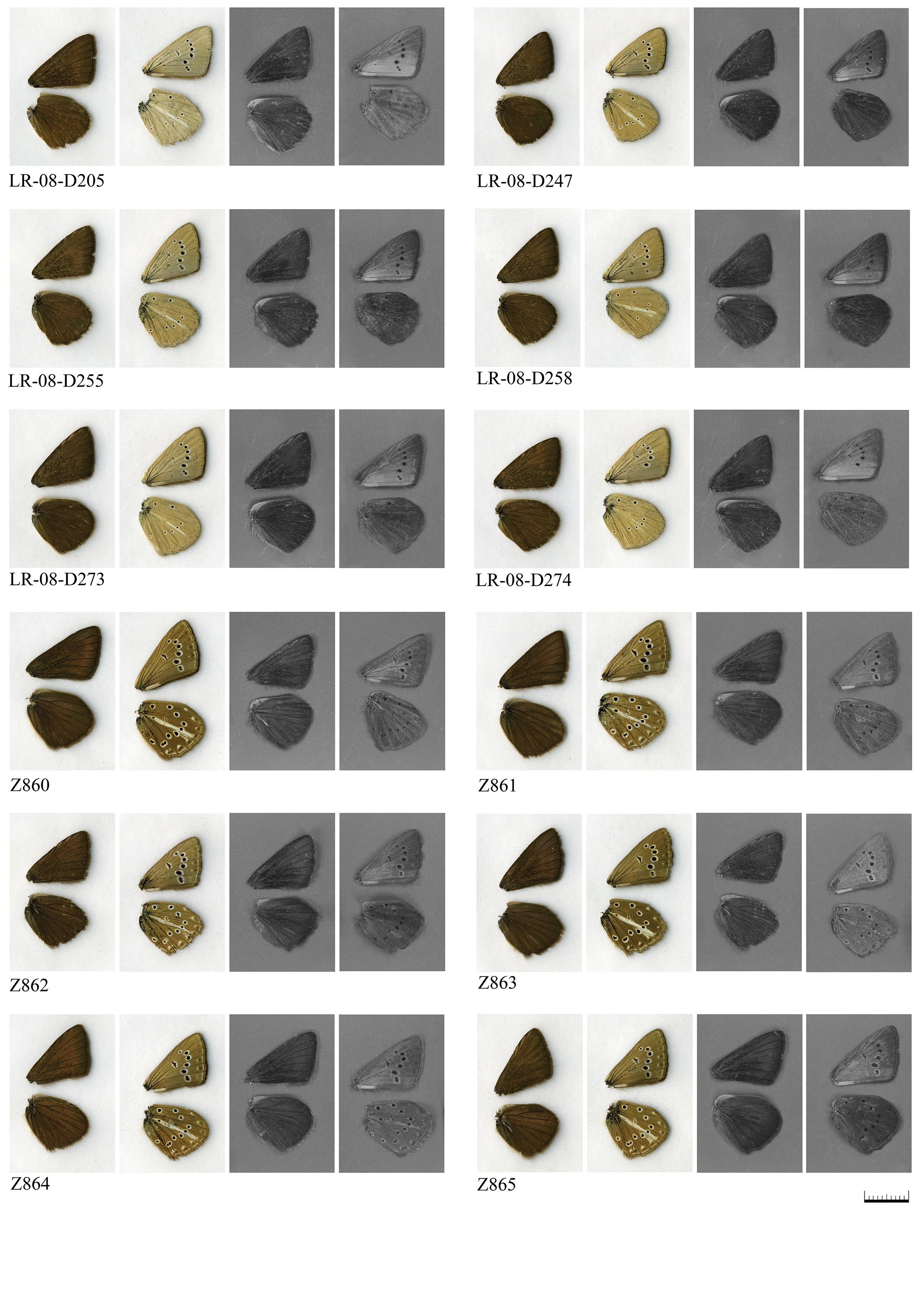

Supplement: Supplementary material 28 — Specimens of P. timfristos and P. valiabadi in visible light and UV light [file zookeys-1256-195_article-165602__-s028.tif]

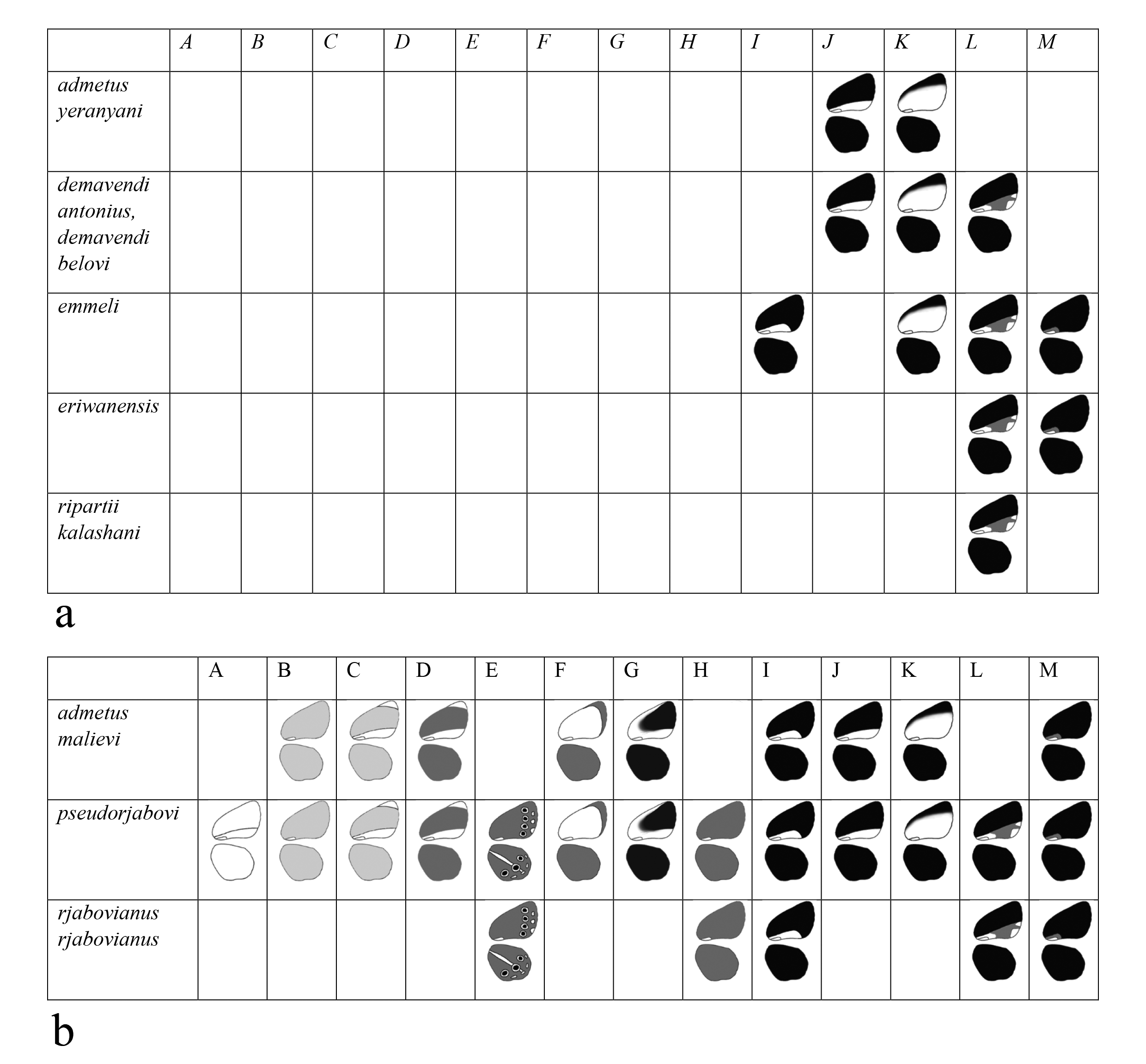

Supplement: Supplementary material 29 — UV pattern analysis. Distribution of UV pattern types in species, inhabiting Armenia and Azerbaijan [file zookeys-1256-195_article-165602__-s029.tif]

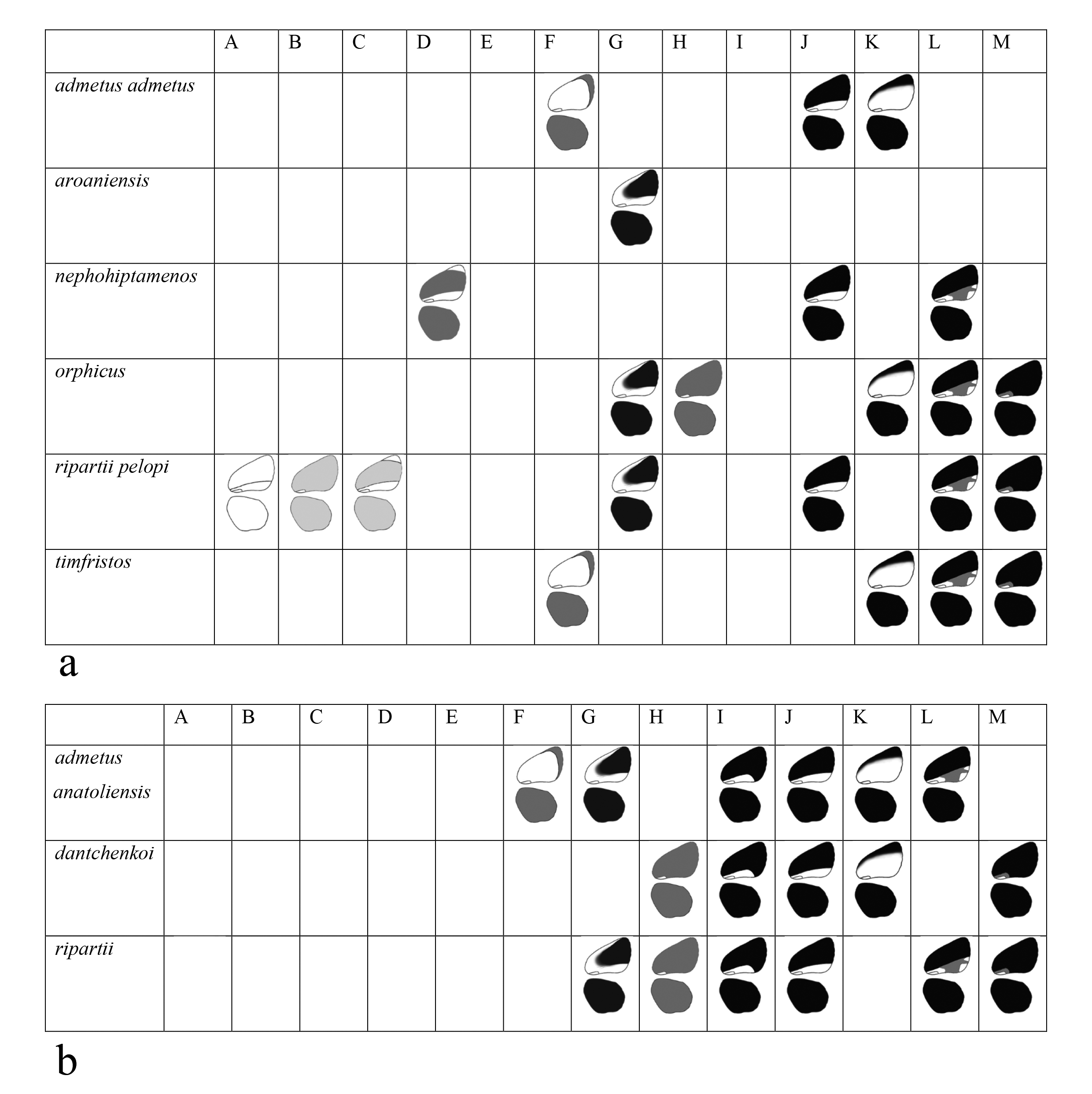

Supplement: Supplementary material 30 — UV pattern analysis. Distribution of UV pattern types in species, inhabiting Balkan Peninsula and Turkey [file zookeys-1256-195_article-165602__-s030.tif]

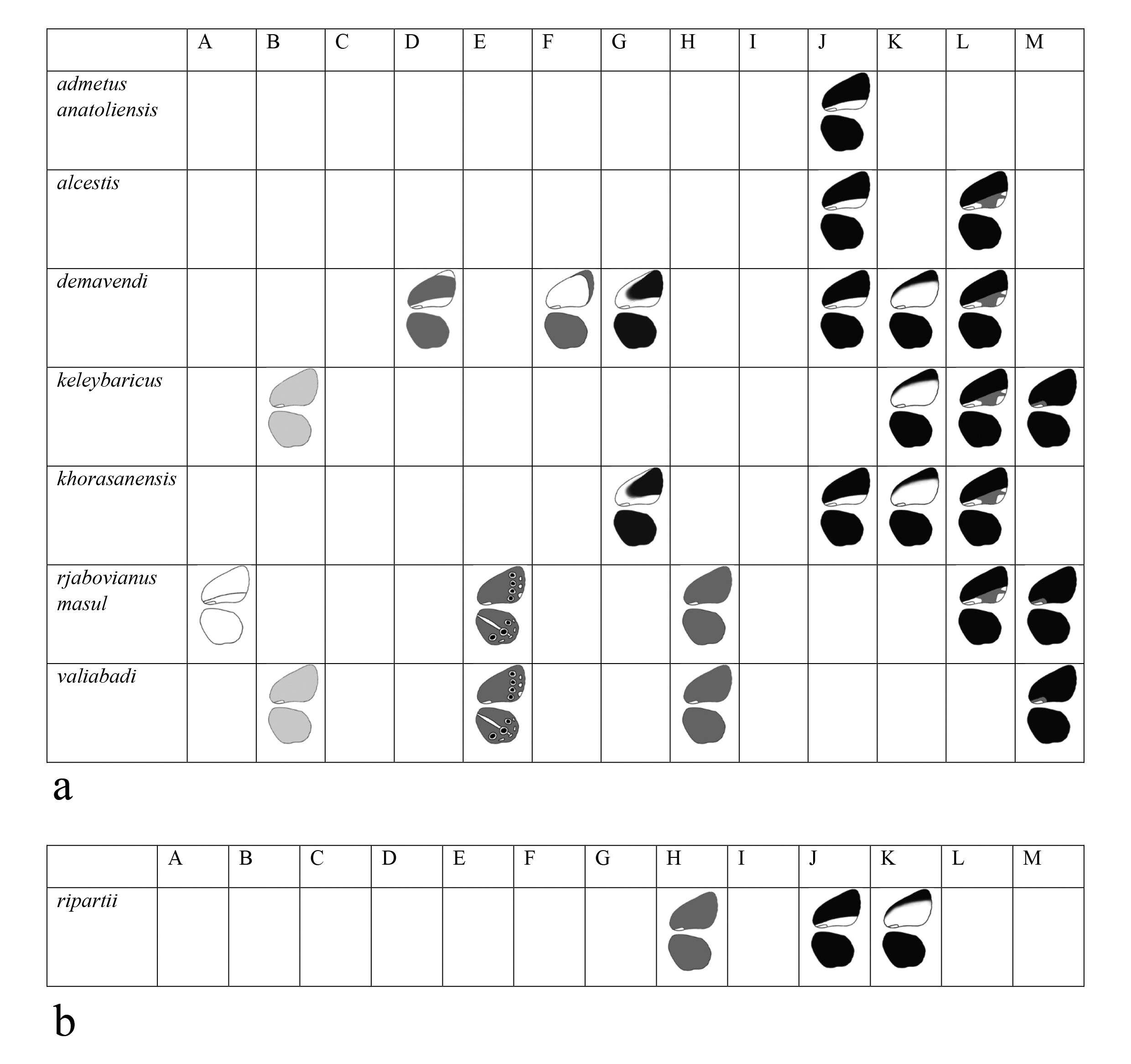

Supplement: Supplementary material 31 — UV pattern analysis. Distribution of UV pattern types in species, inhabiting Iran and Russia [file zookeys-1256-195_article-165602__-s031.tif]

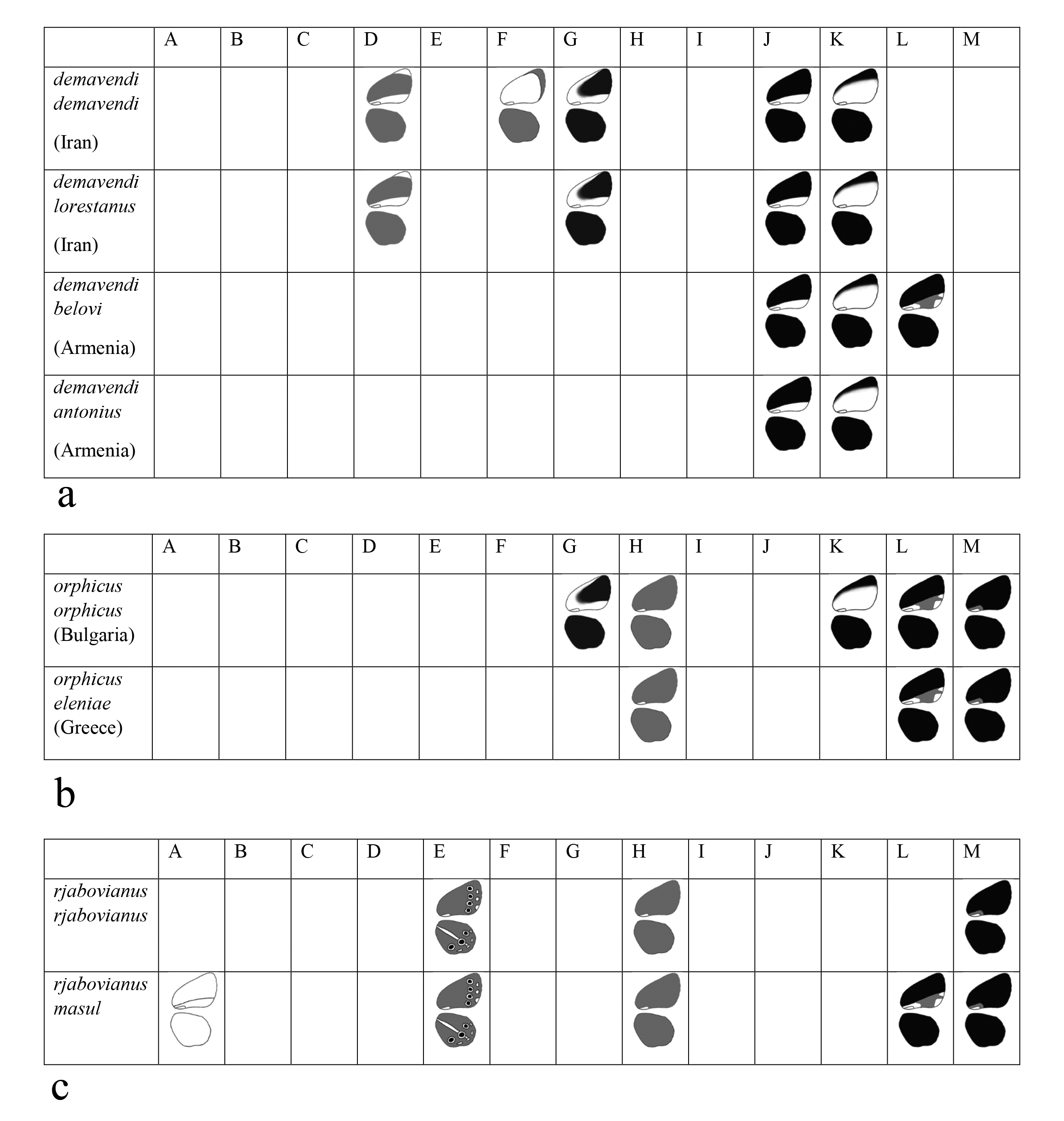

Supplement: Supplementary material 32 — UV pattern types in allopatric populations of one species: P. demavendi, P. orphicus, P. rjabovianus [file zookeys-1256-195_article-165602__-s032.tif]

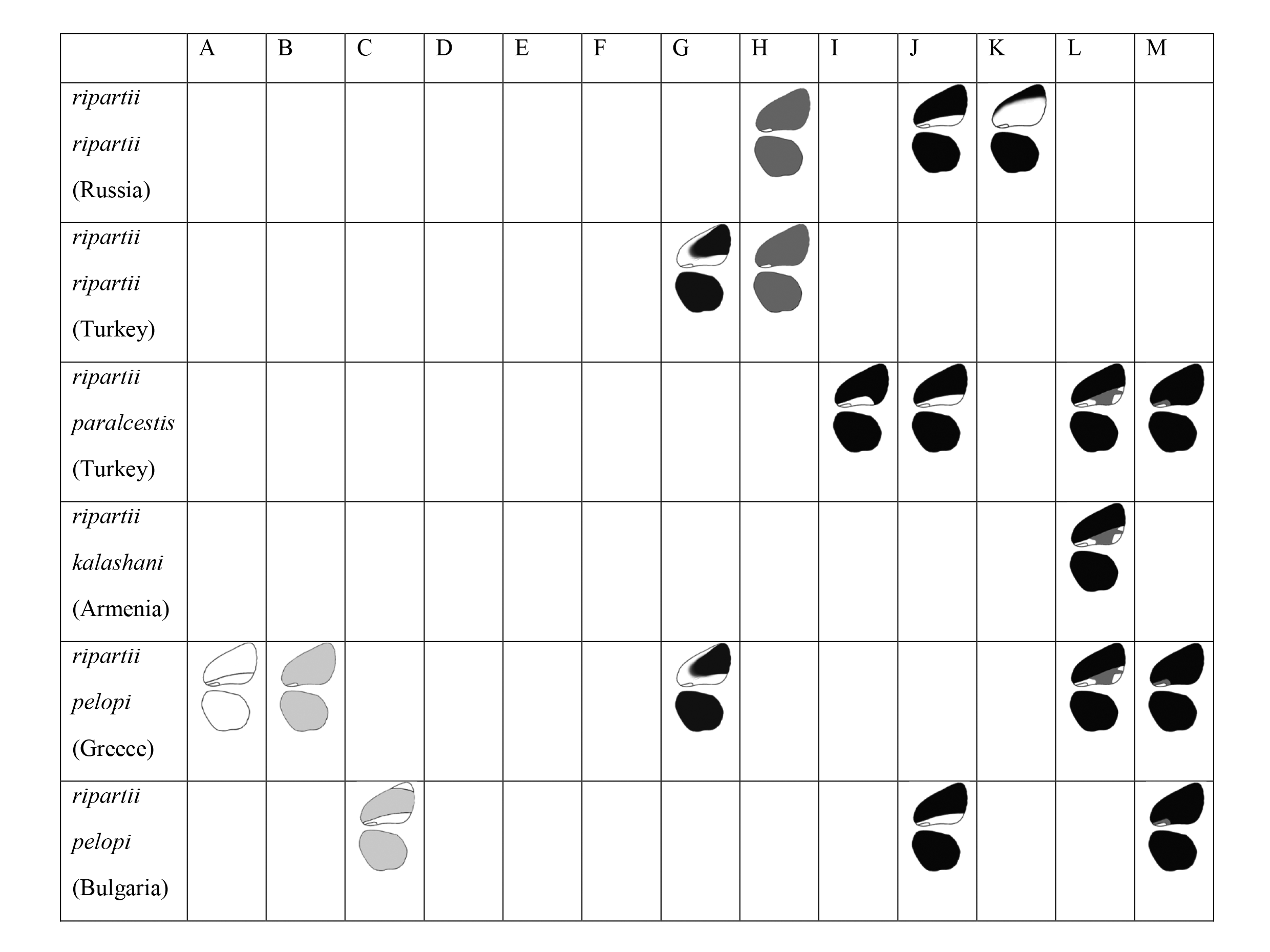

Supplement: Supplementary material 33 — UV pattern types in allopatric populations of one species: P. ripartii [file zookeys-1256-195_article-165602__-s033.tif]

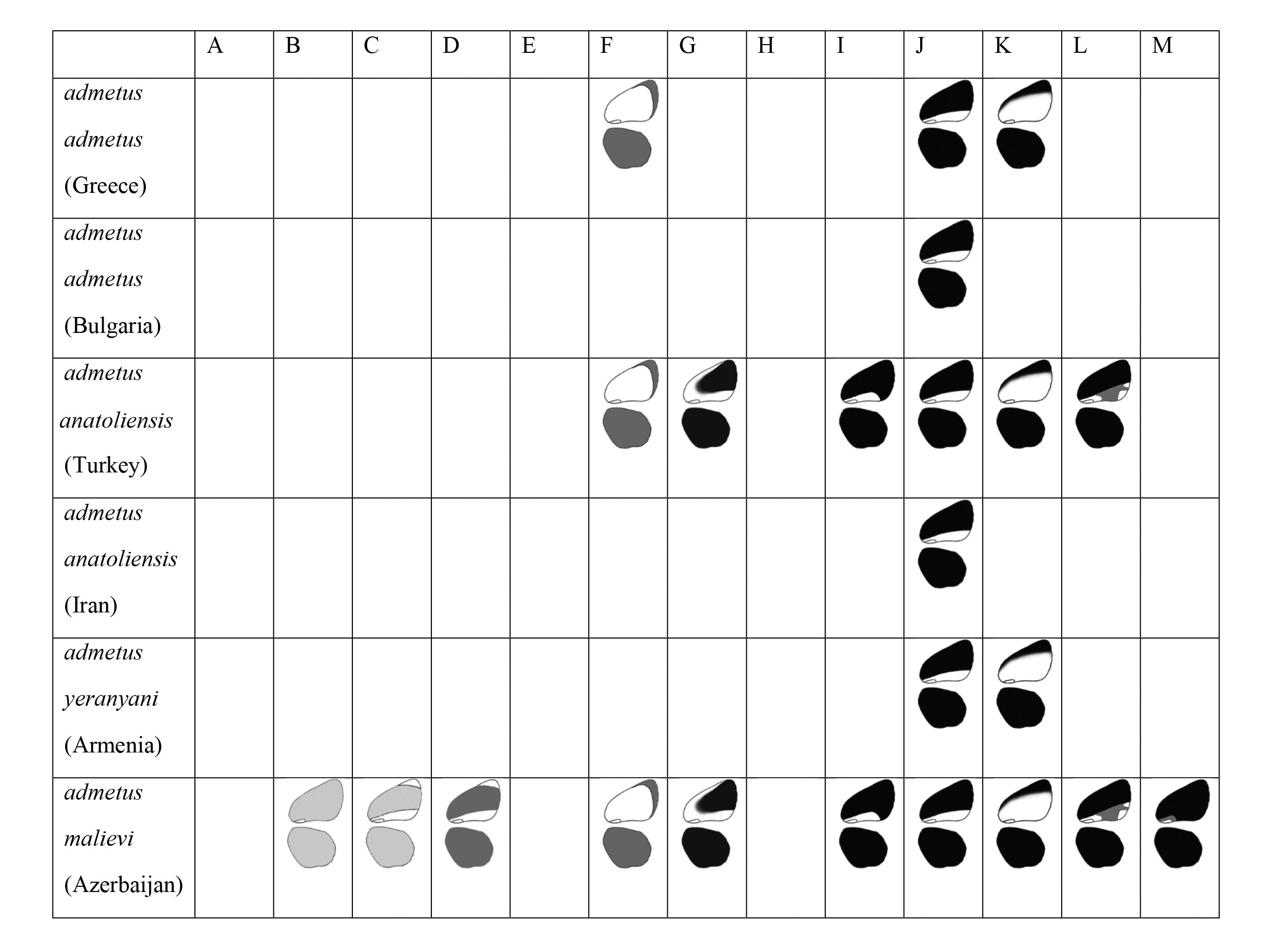

Supplement: Supplementary material 34 — UV pattern types in allopatric populations of one species: P. admetus [file zookeys-1256-195_article-165602__-s034.tif]

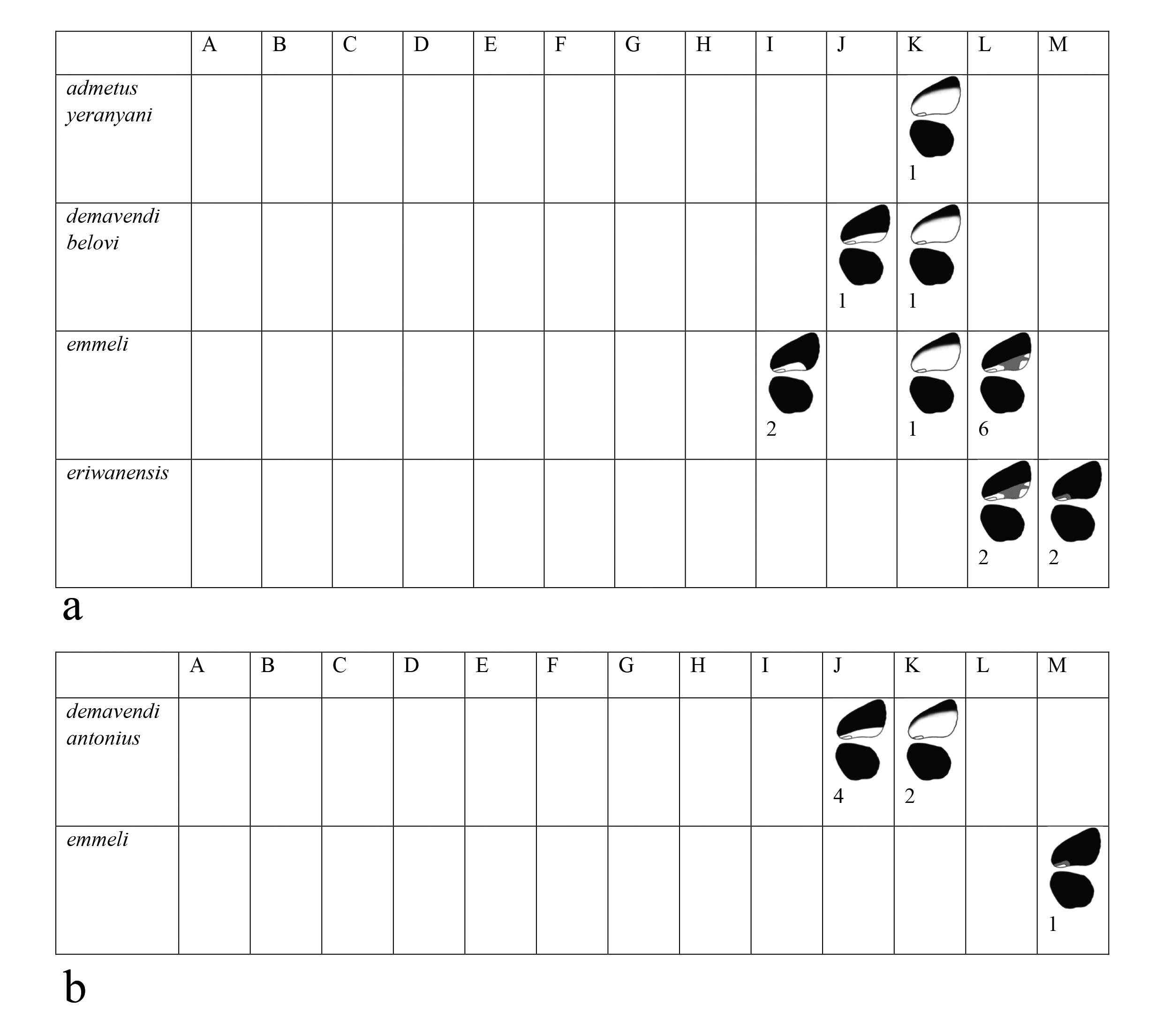

Supplement: Supplementary material 35 — UV pattern types in sympatric species in Armenia [file zookeys-1256-195_article-165602__-s035.tif]

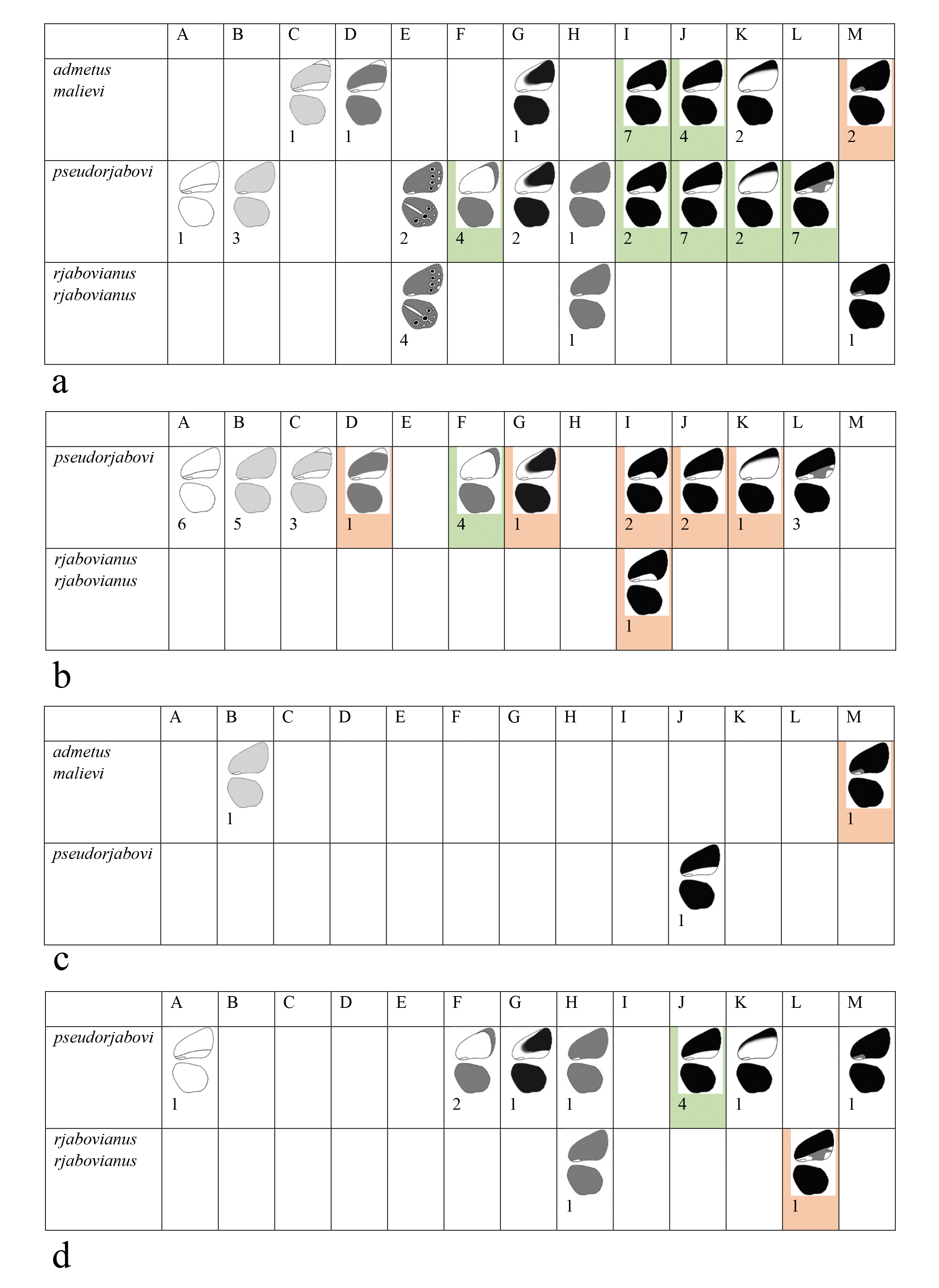

Supplement: Supplementary material 36 — UV pattern types in sympatric species in Azerbaijan [file zookeys-1256-195_article-165602__-s036.tif]

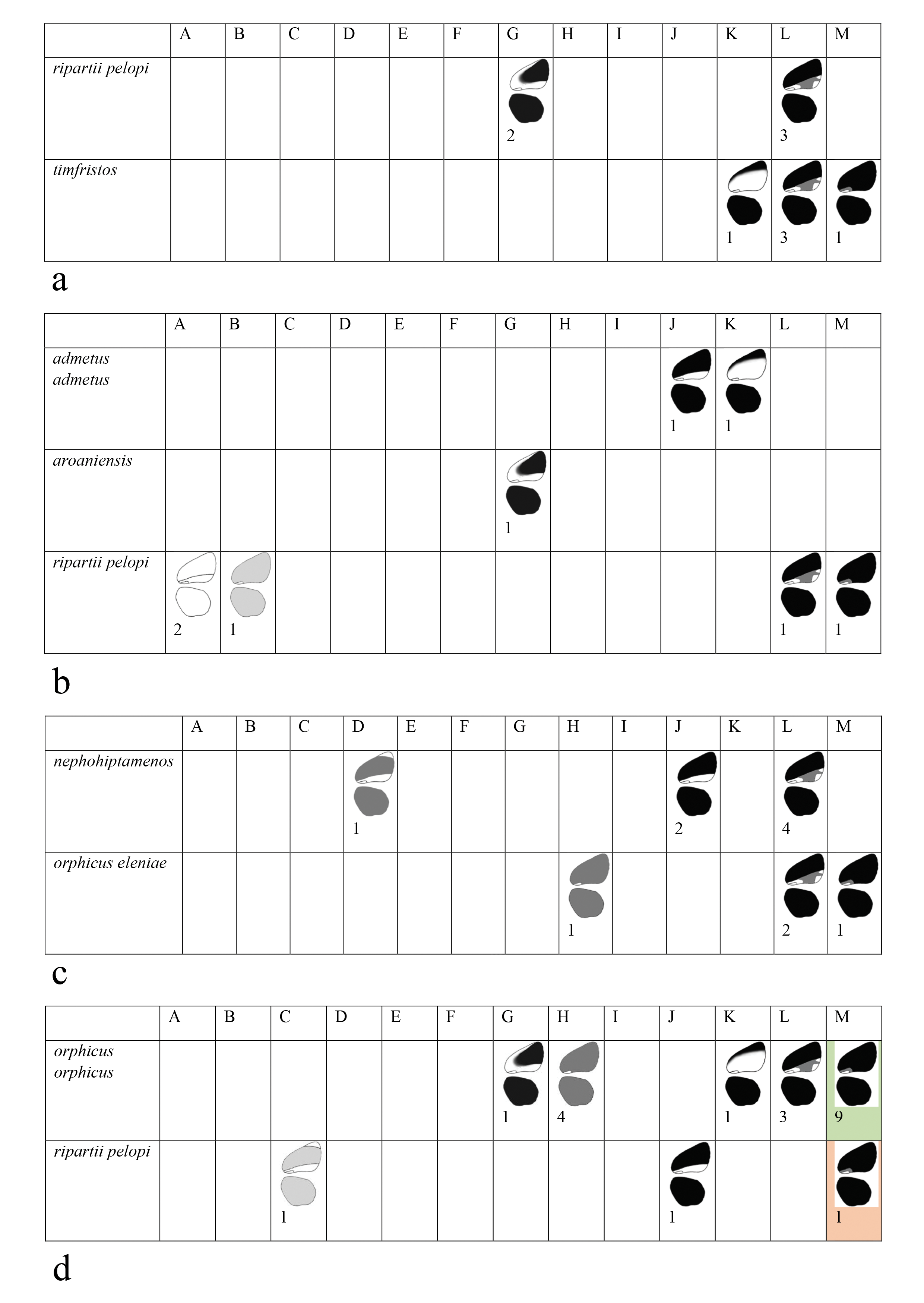

Supplement: Supplementary material 37 — UV pattern types in sympatric species in Balkan Peninsula [file zookeys-1256-195_article-165602__-s037.tif]
